# Jurassic Osmundaceous Landscapes in Patagonia: Exploring the Concept of Ecological Stasis in the Deseado Massif, Argentina

**DOI:** 10.3390/plants14020165

**Published:** 2025-01-08

**Authors:** Juan L. García Massini, Giovanni C. Nunes, Agustina Yañez, Ignacio H. Escapa, Diego Guido

**Affiliations:** 1 Centro Regional de Investigaciones Científicas y Transferencia Tecnológica (CRILAR), Provincia de La Rioja, UNLaR, SEGEMAR, UNCa, Anillaco F5330AGA, La Rioja, Argentina; massini112@yahoo.com.ar; 2 Consejo Nacional de Investigaciones Científicas y Técnicas, Godoy Cruz 2290, Ciudad Autónoma de Buenos Aires C1425FQB, Argentina; cnunes@mef.org.ar (G.C.N.); iescapa@mef.org.ar (I.H.E.); diegoguido@yahoo.com (D.G.); 3 Museo Paleontológico Egidio Feruglio, Avenida Fontana 140, Trelew U9100GYO, Provincia del Chubut, Argentina; 4 Museo Argentino de Ciencias Naturales “Bernardino Rivadavia”, Av. Ángel Gallardo 470, Ciudad Autónoma de Buenos Aires C1405DJR, Argentina; 5 Instituto de Recursos Minerales, Facultad de Ciencias Naturales y Museo, Universidad Nacional de La Plata (UNLP-CICBA), La Plata B1904AMC, Provincia de Buenos Aires, Argentina

**Keywords:** *Osmunda*, wetlands, Deseado Massif, Patagonia, ecological stasis

## Abstract

Herein, we report the presence of a plant paleocommunity, dominated by ferns of the family Osmundaceae, structurally preserved from the only known Mesozoic, fossiliferous geothermal deposits, from the La Matilde Formation (Middle-Upper Jurassic) in the Deseado Massif of Southern Patagonia, Argentina. A total of 13 siliceous chert blocks sampled in an area of approximately 250 m^2^, preserving a monotypic assemblage dominated by Osmundaceae embedded within its original swampy substrate, are documented. Additional Osmundaceae and fewer ferns and conifers are present in the stratigraphically continuous, adjacent chert levels. This association is comparable to those dominated by Osmundaceae in modern swampy settings, such as in high-altitude lagoons in the Paraná Forest in Northeastern Argentina. In addition, a diverse community of mutualistic, parasitic, and saprotrophic microorganisms associated with the ferns and conifers in the assemblage is present. These compositional, paleoenvironmental, and trophic characteristics of the Jurassic Osmundaceae suggest a possible case of ecological stasis, where Osmundaceae-dominated plant communities apparently persisted in swamps of comparable structures, functions, and physical characteristics for over 150 million years. This suggests that Osmundaceae formed similar communities in compatible settings in the Jurassic, becoming preserved in analogous configurations.

## 1. Introduction

The fossil record represents only a fraction of the total biodiversity that has existed across geological times [1]. It is inherently biased and conditioned by the characteristics of the organisms and the depositional environments [1]. However, the fossil record provides qualitative and quantitative information for the reconstruction of the evolution of life and its host environments through time at different hierarchical scales [1].

Fossil preservation occurs in different forms and is influenced by external factors up to the moment of discovery and collection [2,3]. Typically, only parts of an organism are preserved, and it is rare to find whole plants [2,3,4]. In particular, plants are mostly known in the geological record based on isolated organs and sometimes by a couple of organically connected ones [5]. A few very rare examples of exceptional preservation of plants, having even some of their organelles preserved, are known [6]. These unique situations, such as in a *lagerstatten*, provide exceptional scenarios for the reconstruction of organisms, their structure, habits, interactions, and the paleoenvironments they inhabited [6]. In other instances, the reconstruction of whole plants, including their structure, habit, and interactions has been possible by the combined study of isolated and organically connected organs [7].

The possibility of more precise comparisons at the community or ecosystem level with respect to modern environments is more likely for plant lineages with long fossil records [8]. Examples of exceptionally preserved organisms are type cases for ecological and evolutionary studies, such as some plant groups [9,10]. However, even in cases of exceptional preservation, broader ecological aspects of biological communities, such as spatial arrangement, physical structuring, and use of morphspace through time, are only possible to examine in uniquely preserved paleoenvironments. Another challenge in interpreting ancient plant communities is the taxonomic compositional differences between these ancient ecosystems and the modern equivalents [3], which slows the reconstruction of entire ecosystems and limits precise comparisons with modern environments.

Recognition of these unique paleontological situations in the fossil record is most unusual depending upon multiple factors, including the preservation of the organisms in life position within their original substrate, which provides a unique opportunity to reconstruct the ecological history of biotic communities and their ecosystems across geological times [3]. Examples of this in the geological record can be detected from the conservation of community structure and the morphological characteristics of the fossil taxa with modern representatives, within their original paleoecosystems, and are suggestive of ecological stasis [11].

Ecological stasis refers to those moments in the paleontological record where biotic communities and their environments remain ecologically and morphologically stable across geological time [11]. In those cases, evolutionary processes are centered on the ecosystem organization resulting from the structuring of biological communities, which show a resilient tendency to face disturbances [11]. This selection via evolutionary processes on populations is, at the same time, constrained by their influence at the ecosystem scale, where its effect is less prominent, deriving from higher ecological stability. A fluctuating equilibrium occurs, where different expressions of analogous conformations for a given community replace each other across time [12]. However, sometimes this equilibrium is interrupted by a disturbance that drives their change or evolution to, eventually, their extinction or to their modern forms [11].

The family Osmundaceae Martinov is a stereotypical case of conserved morphological and ecological characteristics, especially since the Mesozoic [5,8,13]. Its general body plan, habit, metabolic pathway, and even, apparently, its genetic configuration are perhaps the most relatively conserved ones among ferns [6,9,14,15]. Some of this apparent conservation has been questioned, however, and is indicated to be an incomplete reflection of the real variation available from studying only fossils [16]. Despite this, its fossil record is abundant and constant since the Permian, including numerous morphospecies from Patagonia and worldwide [5,17,18,19]. It is a monophyletic clade that represents a distinctive line of evolution, whose abundance has apparently shrunk after the Mesozoic and gave rise to new forms during the Cenozoic, but which remained basically morphologically similar throughout [5,20]. The observed history of morphological and molecular stability for Osmundaceae based on its extensive fossil record is also apparent in its ecology [5]. Osmundaceous fossils have been described from wet, tropical-to-temperate paleoenvironments, which is also what, generally, their modern habitats are, although they are variable with respect to their distribution and other environmental aspects [5,21]. However, the majority of research papers carried out focused on taxonomic studies of single organs and phylogenetic analyses of selected fossil and extant species [14,22,23]. The study of preserved fossil communities has been hindered by the lack of examples, and as far as we found, in situ and in-life position examples of Osmundaceous are unknown from the fossil record. Herein, we describe a plant assemblage dominated by the fern family Osmundaceae, accompanied by a diverse microbial community associated with the ferns and subordinate plants, embedded in life within its original swampy substrate from a Jurassic geothermal landscape in Southern Patagonia, Argentina. This unique example provides information on the composition, structure, and network of interactions within a Jurassic swamp in Patagonia, which allows for characterizing the paleoecosystem and evaluating its similarity to Osmunda-dominated, modern analogous ecosystems.

## 2. Materials and Methods

**Geological context and paleontological content of the studied area, La Bajada.**

The fossil material was collected from outcrops of the La Matilde Formation at La Bajada locality in the Deseado Massif. The Deseado Massif is a geological province with an approximately 60,000 km^2^ extension, located in the Santa Cruz Province of Southern Patagonia, Argentina [24,25]. The geological history of the area is characterized by a bimodal rhyolitic and andesitic volcanic event that led to the formation of the Bahía Laura Complex approximately 178–151 million years ago [26,27]. These rocks are part of the Chon Aike Silicic Large Igneous Province that extends from Argentinean Patagonia to Antarctica [28] and whose genesis is associated with the break-up of Gondwana and the opening of the South Atlantic Ocean [29,30].

**Figure 1:**
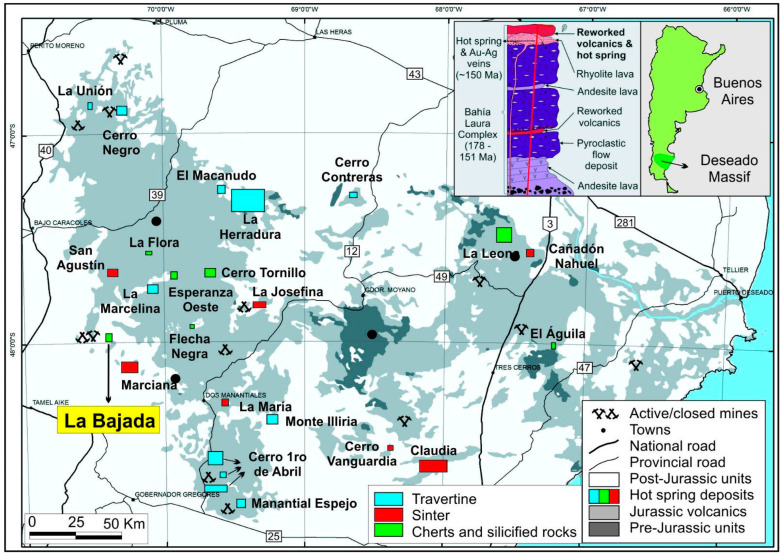
Map of the Deseado Massif (Santa Cruz Province, Argentina) showing the distribution of the Jurassic geothermal deposits and the associated fossiliferous chert localities, indicating the location of the studied area and stratigraphic sequence at the La Bajada locality.

**Figure 2:**
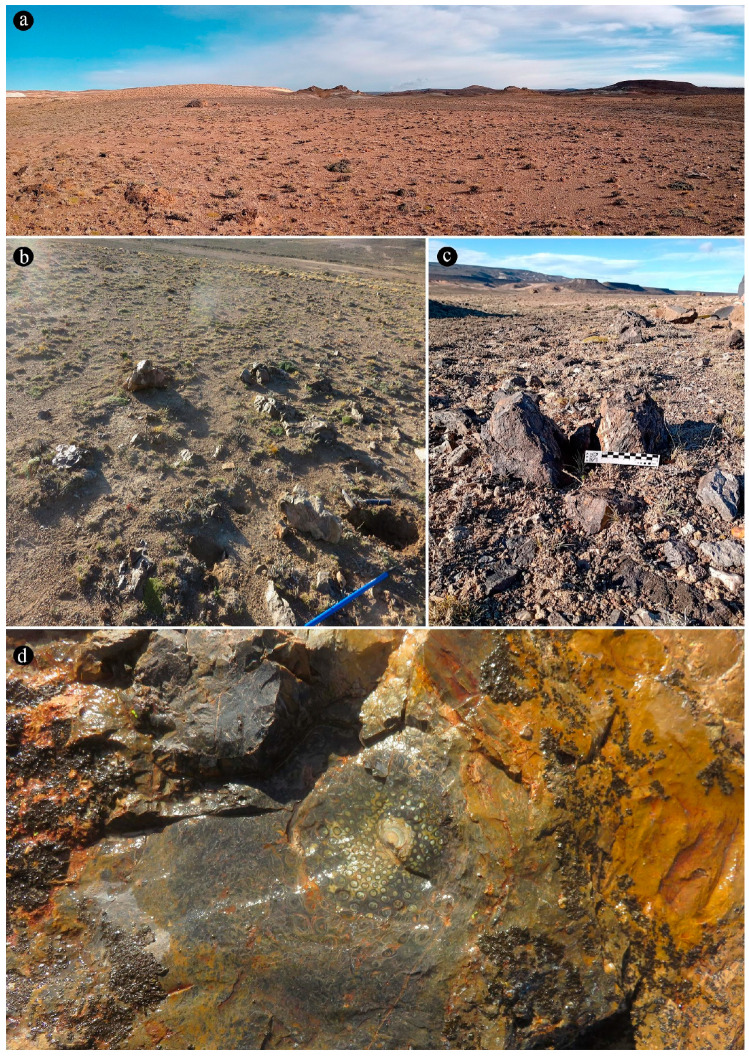
Studied area and distribution of in situ plants at the La Bajada locality, Santa Cruz Province, Argentina. (**a**) General view of the studied area at the La Bajada locality where the geothermal deposits outcrop. (**b**) Distribution and excavation of chert blocks bearing numerous osmundaceous ferns in life position. (**c**) Cluster of semi-buried chert blocks with in situ fern stems. (**d**) Detail of in situ fern stem embedded in the chert bed in transverse section.

The La Bajada locality (Figure 1 and Figure 2) is located in the western portion of the Deseado Massif Jurassic volcanic outcrops [25], and it is characterized by siliceous hot-spring-related deposits hosted in reworked volcaniclastic sediments of the La Matilde Formation [25]. The rocks at ‘La Bajada’ show the typical association of geothermal features with volcaniclastic fluvial or lacustrine reworked deposits and volcanic lava domes (lithological control proposed by [24]). A series of fossiliferous sedimentary chert beds outcrop irregularly in a 2.5 × 0.5 km area in close proximity to the La Bajada farmhouse [25]. These rocks vary from strongly silicified volcaniclastics to pure cherts deposited in a distal, geothermally influenced marsh facies setting [25,31].

Preliminary analyses of the chert samples from La Bajada hot-spring chert deposits offered a first glimpse of an exceptionally preserved biota [25] composed of different groups of animals, plants, and a broad range of microorganisms [25,32,33]. Along with the vascular plants, there are abundant vegetative and reproductive fern remains with different systematic affinities [25], including the rhizomatous stems assigned to the Osmundaceae species *Millerocaulis zamunerae* Sagasti et al. [18]; pinnules, sori and isolated sporangia of putative Gleicheniaceae C.Presl, Cyatheaceae Kaulf., and Dicksoniaceae M.R.Schomb. [25]. Conifer vegetative and reproductive remains with araucarialean affinities are also common in these cherts, including wood fragments, roots, leafy twigs, and cones with pollen in situ [25,32,33]. Further plant remains include stems of *Equisetum thermale* Channing et al., which are frequently fragmentary [25]. The microcommunities are characterized by different filamentous and colonial cyanobacteria; filamentous and unicellular algae; abundant peronosporomycetes with combresomycetelean affinities; testate amoeba and ciliates; and very diverse and abundant fungi [25,32,33,34]. Different systematic and ecological groups of fungi are frequently associated with different plant remains, and are represented by abundant vegetative and reproductive remains [25], including epibiont chytrids, mycorrhizal glomeromycetes, epiphyllous ascomycetes, and a variety of ascomycetes and basidiomycetes [25,32,33,34]. Furthermore, remains of terrestrial and freshwater invertebrates are also present (e.g., appendages, mouthparts, abdomens, and compound eyes of arthropods; ostracod and other crustaceans carapaces; gastropod shells; worms, rotifers, nematodes, and coprolites of various morphologies) [25].

**Preparation and imaging of fossil material.** Several chert blocks were cut following standard techniques [35] and using two different Hillquist (Denver, CO, USA) slab saw machines, one for the larger rock samples and one for the hand-sized rock samples. Some blocks were cut in several sections, following the longitudinal axis of the plant individuals to obtain serial planes and ensure the exposure of as much surface area as possible to observe the surrounding matrix. The chert blocks and their sections were observed using a Zeiss (Oberkochen, Germany) MC80DX and a Nikon (Tokyo, Japan) SMZ1000 stereoscope. Additionally, thin sections of some of the chert samples were prepared following standard methodology [36] and using a Buehler petrothin sectioning machine. Slides were observed using transmitted light microscopy. 

**Figure 3:**
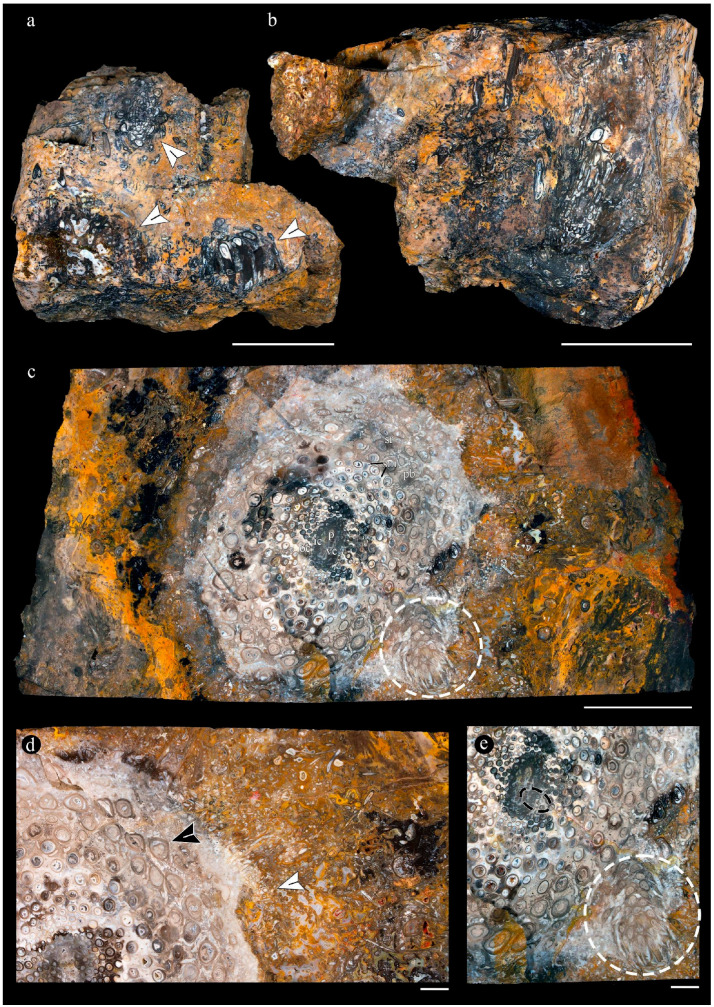
Fossil Osmundaceae Martinov. (**a**) Cross section of rhizomes of three individuals (white arrows). (**b**) Longitudinal section of the rhizome. (**c**) Cross section of the rhizome showing internal petiole cycles and root mantle. White dotted circle indicates a lateral rhizome separating from the main plant for vegetative reproduction. Notice the central stem comprising the collapsed pith (p), vascular cylinder (vc), parenchymatous inner cortex (ic), and sclerotic outer cortex (oc); and the outer cycles of petiole bases (pb) characterized by stipular wings with sclerotic masses (sm) and a sclerotic ring (sr). (**d**) Detail of (**c**) showing petioles (black arrow) close to the central cylinder and superficial roots (white arrow). (**e**) Detail of (**c**) showing lateral rhizome (white dotted circle). Observe the formation of the leaf trace (black dotted ellipse), which separates from the xylem cylinder with only one protoxylem cluster, which divides in the outer cortex. Bar = 5 cm, (**d**,**e**) 1 cm.

**Figure 4:**
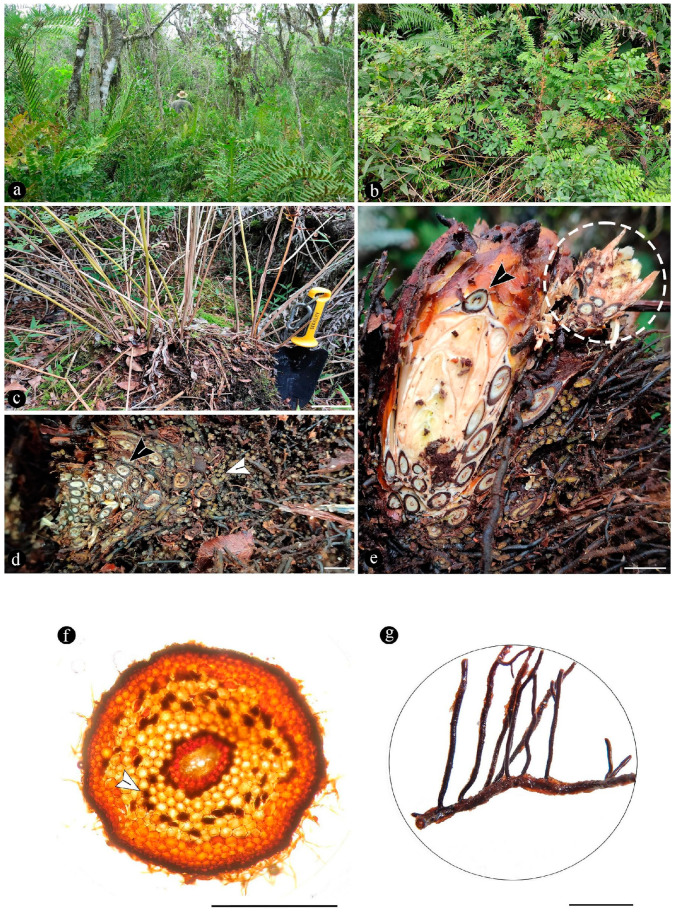
Extant *Osmunda spectabilis* Willd. in wetlands from Misiones province. (**a**) Wetland dominated by *O. spectabilis* and *Neoblechnum brasiliense* (Desv.) Gasper and V.A.O.Dittrich. (**b**) General view of *O. spectabilis* plants. (**c**) Exposed underground rhizome showing the emergence of densely arranged petioles. (**d**) Cross section of the rhizome showing petioles (black arrow) close to the central cylinder and superficial roots (white arrow). (**e**) Cross section of the rhizome showing petioles (black arrow). A dotted circle indicates a lateral rhizome separating from the main plant for vegetative reproduction. (**f**) Cross section of roots. White arrow indicates colonization by arbuscular mycorrhizae. (**g**) Surface view of branched roots of different diameters. Bar = (**c**) 4 cm, (**d**,**g**) 5 cm, (**e**) 8 cm, (**f**) 150 μm.

**Figure 5:**
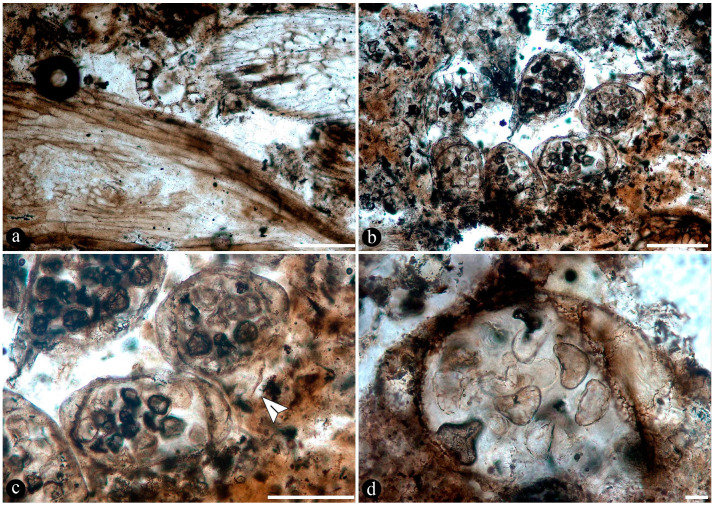
Fossil sporangia embedded in the chert matrix, associated with the rhizomatous stems. (**a**) General view of chert matrix containing roots belonging to the Osmundaceae Martinov. Rhizomatous stems, showing associated remains of the annulus of a single sporangium, composed of thick-walled cells. (**b**). General view of group of sporangia with variable preservation with in situ spores, preserved along the rhizomatous stems. (**c**) Close-up of some of the sporangia, showing the presence of a short stalk (white arrowhead). (**d**) Close-up of a sporangium containing numerous trilete spores. Bar = (**a**,**b**) 1 mm, (**c**) 70 μm, (**d**) 20 μm.

**Figure 6:**
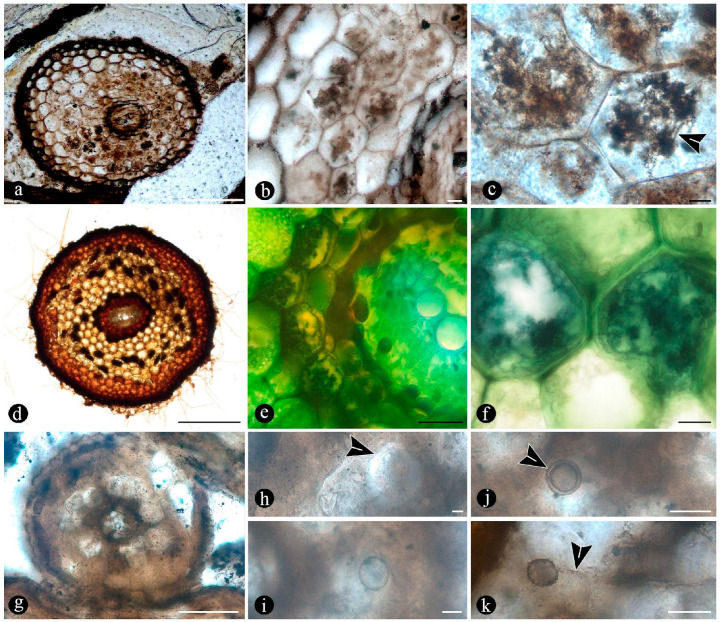
Fungal interactions in the roots of fossil and extant Osmundaceae Martinov. (**a**) General view of silicified adventitious diarch roots of rhizomatous stems of fossil Osmundaceae preserved together in a siliceous chert matrix, formed around an in situ community in geothermal settings from the Jurassic of Patagonia, Argentina. (**b**) Detail of small diarch fossil root filled with fungal material, especially cortical cells around the pericycle. (**c**) Close-up on a few cortical cells showing fungal material consisting of highly ramified arbuscules. Note (arrowhead) arbuscules emerging from broader, trunk hypha. (**d**) Detail of extant *Osmunda spectabilis* Willd. small diarch root filled with fungal material. (**e**) Close up of cortical cells of living material of *O. spectabilis* diarch root showing distribution of fungal material around the pericycle. (**f**) Close up on a few cortical cells showing highly ramified arbuscules in living material of *O. spectabilis* small diarch root. (**g**) Another diarch root with cortical cells filled with fungal material and an amorphous residue. Also, note some broken cells and the resulting cell-free area to the upper right of the root. (**h**) Detail of (**g**) showing coenocytic hyphae (arrowhead) within some cortical cells. (**i**) Detail of (**g**) showing globose, intracellular spore. (**j**) Close-up on cortex of (**g**) showing another, 2-layered intracellular spore, subtended by a short hypha. (**k**) Globose spore in the matrix around (**g**) and connected to it by a long subtending hypha (arrowhead). Bar = (**a**) 200 μm, (**b**,**f**,**i**–**k**) 20 μm, (**c**,**h**) 10 μm, (**d**) 500 μm, (**e**) 65 μm, (**g**) 150 μm.

**Figure 7:**
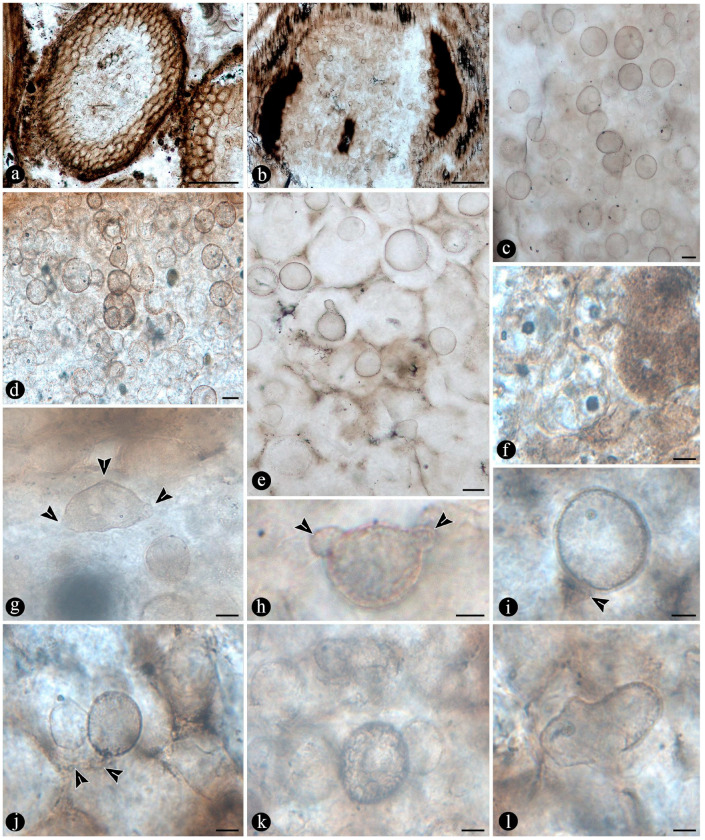
Diversity of saprotrophic and parasitic microorganisms in decayed petioles and roots of Osmundaceae material. (**a**) Decayed adventitious diarch root of fossil Osmundaceae Martinov. with a very large number of globose structures distributed in the partially hollowed-out tissues. (**b**) Decayed petiole of Osmundaceae also with a large number of associated globose structures within the degraded tissues. (**c**) Close up on (**b**) showing spherical to pyriform to globose structures. (**d**) Additional globose structures of variable size, texture, and morphology included in (**b**). (**e**) Close up on decayed cells containing a single endobiotic globose structure. (**f**) Close up on other cortex cells with several endobiotic, smaller globose structures. (**g**) Detail of a bigger globose structure with 3 symmetrically arranged, somewhat protruding pores (arrowheads). (**h**) Detail of another globose structure showing 2 symmetrically arranged, dome-like apertures (arrowheads). (**i**) Detail of a globose structure with a faintly granulated surface and a somewhat tapered apophysis (arrowhead). Note also its relatively thicker wall formed by at least two layers. (**j**) Close up of another globose structure, attached to the host tissues with a finely granulated thread of minute hyphae or rhizoids (arrowheads). (**k**) Detail of another globose-to-pyriform structure showing a granulated cytoplasm consisting of small circular to quadrangular subunits. Note also its relatively thicker wall formed by at least two layers. (**l**) Close up on another globose to amoeboid, collapsed structure, partly disrupted and containing a few hyaline smaller globose structures. Bar = (**a**,**b**) 250 μm, (**c**–**l**) 15 μm.

**Figure 8:**
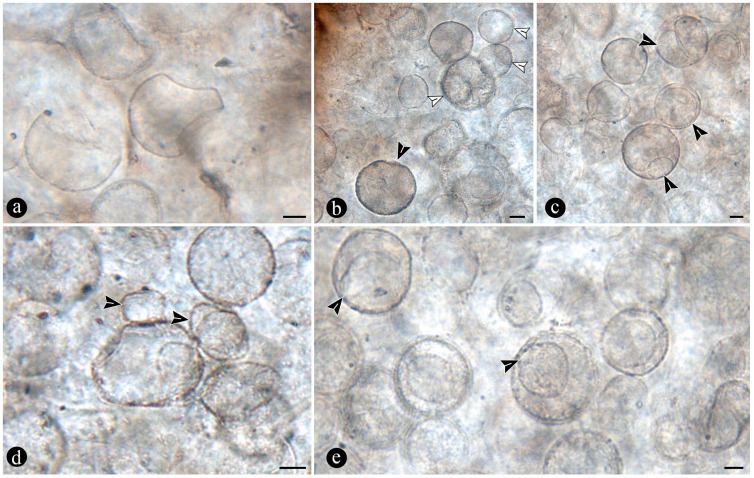
Diversity of saprotrophic and parasitic microorganisms in decayed petioles and roots of Osmundaceae material. (**a**) Close up on a group of globose structures with bowl-like and tote-bag-like parts of their bodies missing. (**b**) Group of globose structures, also with some having a shallower section of their bodies missing (black arrowhead) and with epibiotic globose structures attached to their outer surfaces (white arrowheads). (**c**) Detail of additional globose structures with endobiotic globose structures (arrowheads). (**d**) Another globose structure with several epibiotic ones (arrowheads) attached to its outer surface. (**e**) Group of globose structures with a single endobiotic globose structure, partly to almost completely filling their lumina, and with a short neck that extends through the wall of the host to the exterior (arrowheads). Bar = (**a**–**e**) 15 μm.

**Figure 9:**
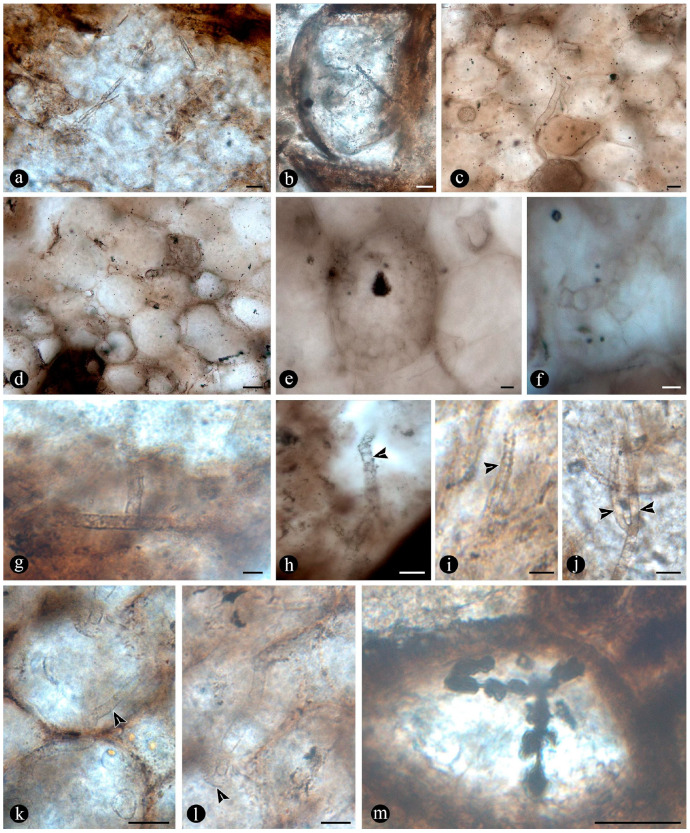
Diversity of saprotrophic and parasitic microorganisms in decayed petioles and roots of Osmundaceae. (**a**). Filaments in a degraded petiole of the fossil Osmundaceae Martinov. (**b**) A decayed fern sporangium with thin, septate filaments. (**c**) Cortical cells of a fern petiole with inter and intracellular, broad, mostly coenocytic filaments. (**d**) Cortical cells of a fern petiole with filaments of variable width, distributed in the cellular interspaces and intracellularly. (**e**) Close up on a cortical cell, which is entirely filled by highly contorted, broad filaments that intersect each other, resulting in a reticulate pattern. (**f**) Cortical cells of a fern petiole with closely septate, broad, intracellular filaments consisting of a series of consecutive cylindrical units. (**g**) Close up on degraded tissues with branched, septate filaments. (**h**) Close up on a branched filament in degraded tissue. Note (arrowhead) septation of the hypha, which gives rise to consecutive cylindrical cells at the distal end of the lateral branch (arrowhead). (**i**) Another example of branched filament in degraded fern tissues, divided into disc-like cells (arrowhead), at the distal end of the lateral branch. (**j**) Close up on a filament consisting of a principal filament with two symmetrical upright branches (arrowheads). (**k**) Close up on another filament with a single upright branch (arrowhead). (**l**) Close up on a filament with upright branches that is attached to the host substrate through a broad base (arrowhead). (**m**) Smaller, opaque, branched filaments displaying branches with consecutive small globose cells within the lumina of cortical cells of a fossil petiole. Note lateral branches further subdivided into small subunits in a rosary-like pattern. Bar = (**a**,**b**) 30 μm, (**c**–**f**) 15 μm, (**g**–**m**) 10 μm.

**Figure 10:**
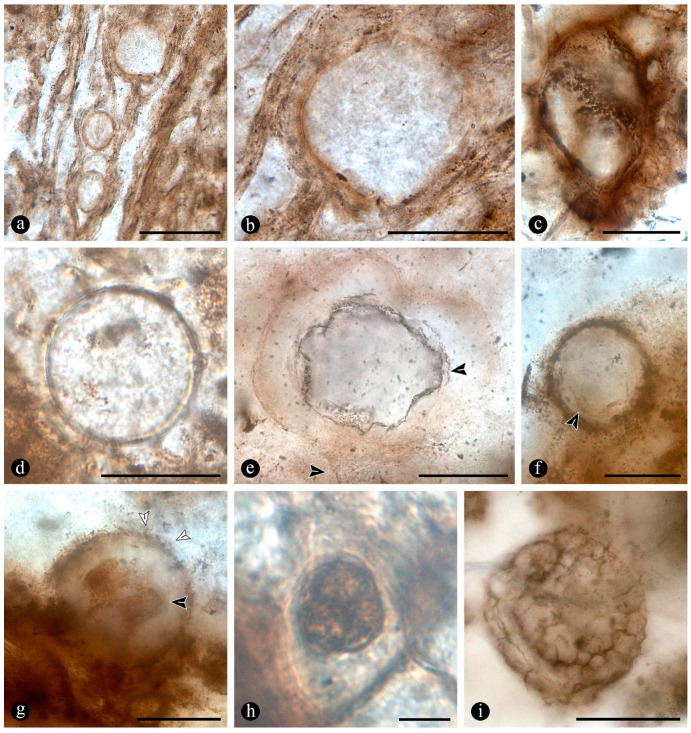
Diversity of saprotrophic and parasitic microorganisms in decayed petioles and roots of Osmundaceae. (**a**) Globose-to-elliptical structures of different sizes, with characteristic thick and layered walls, encysted in degraded fern tissues. (**b**) Close up on a pyriform structure encysted in decayed tissues with a multilayered wall and possible opposite broad apertures at the poles. (**c**) Endobiotic elliptical structure associated with cortex cells of fossil petiole l. Note its thick wall and granulated contents. (**d**) Structure of circular section found in the matrix surrounding the rhizomatous stems. Note its characteristic thick wall and approximately trapezoidal base of external ornaments. (**e**) Broadly pyriform structure within decayed fern tissues, with a thick and multilayered wall. Note the presence of a centrally placed, hyaline structure (arrowhead) and broad subtending hypha (arrowhead). (**f**) Globose, thick-walled structure ornamented with short spines embedded within degraded organic debris. Note central structure (arrowhead). (**g**) Globose structure with spiny ornamentation (white arrowheads), encysted in decayed organic debris. Note the opaque, central structure (arrowhead). (**h**) Helicoidal, multicelled fungal spore associated with decayed fern tissues. (**i**) Multicelled dictyospore preserved within decayed fern tissues. Bar = (**a**) 100 μm, (**b**–**g**) 50 μm, (**h**) 10 μm, (**i**) 25 μm.

**Figure 11:**
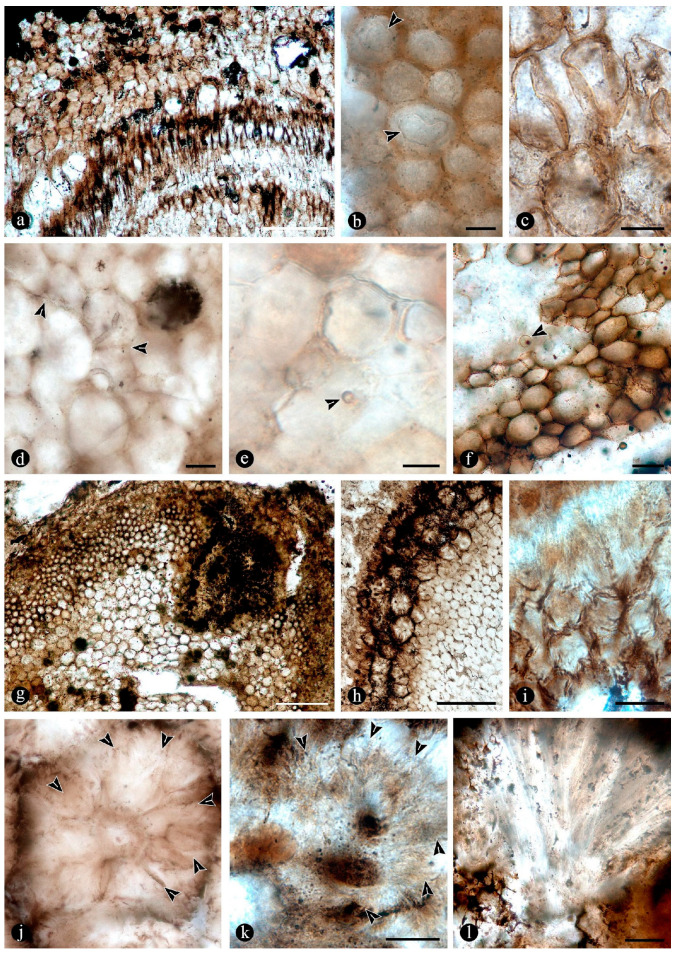
Decay patterns of degraded petioles and roots of fossil Osmundaceael. (**a**) General view of degraded fern fossil petioles having deformed and variously disrupted cells, sometimes filled with an opaque substance. (**b**) Close-up on degraded cortex cells, including some with their innermost cell component of the secondary wall (i.e., S3) disrupted and projected inwards into the lumen (arrowheads). (**c**) Close-up on another group of cortex deformed and distorted cells showing differential decay of the lignin-rich cell wall components (i.e., middle lamella, primary wall). (**d**) Close-up on another group of degraded petiole cells showing decay of all cell wall components, which remain connected through their lignin-rich junctions at the cell corners (arrowheads). (**e**) Close-up on additional degraded petiole cells showing decay of all cell wall components and filaments (arrowhead) in direct contact with the decayed cells. (**f**). Decayed fossil petiole with small globose structures (arrowhead) attached to the disrupted tissues. (**g**) Decayed irregular area with a characteristic fuzzy texture. (**h**) Another fern petiole showing a cluster of smaller, regular, approximately circular decay areas, with a characteristic furry, radiating texture. (**i**) Close-up on another decayed area showing distorted and eroded cells with smooth textures formed by filamentous-like features. (**j**) Close-up of another decayed area showing cells with smooth, velvety-like textures and a radiating appearance (arrowheads). (**k**) Close-up on another fuzzy decay area with even less recognizable cells, also having decay features with velvety or silky textures with a radiating arrangement (arrowheads). (**l**) Close-up of another fuzzy decay area where individual cells are not recognizable, having an even more silky texture and cell remains arranged in a pattern consisting of radially oriented, fusiform features. Bar = (**a**,**g**,**h**) 250 μm, (**b**–**e**) 25 μm, (**f**,**i**–**l**) 50 μm.

**Figure 12:**
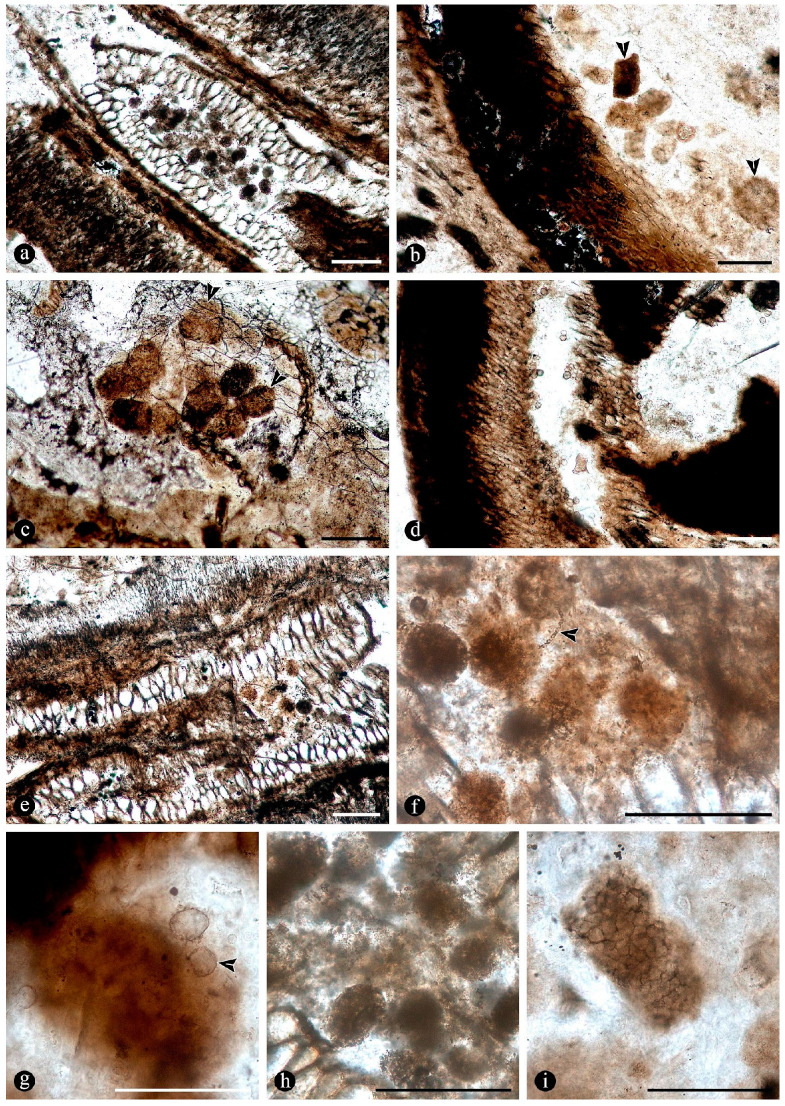
Evidence of mesofauna associated with degraded petioles and roots of fossil Osmundaceae. (**a**) General view of degraded fern fossil petiole centered in the vascular trace area, which is partly decayed and filled with clusters of small coprolites. (**b**) Another degraded fossil petiole with bigger coprolites (arrowheads) distributed within the degraded tissues of the cortex. (**c**) Highly degraded adventitious root filled with big cylindrical coprolites (arrowheads). (**d**) Boring patterns in a degraded fossil petiole consisting of an extensive irregular decay area with all tissues consumed away. (**e**) General view of another decayed fern petiole having an opaque, amorphous, organic residue and some small coprolites in the degraded area. (**f**) Close up on a group of small coprolites included in a degraded fern petiole formed of highly comminuted particles and a smooth texture. Note the presence of a filament (arrowhead) directly associated with the coprolites. (**g**) Close up on a big coprolite associated with the degraded fern petioles showing globose structures attached to its surface (arrowhead). (**h**) Close up on a cluster of small coprolites with a characteristic elliptical shape and circular cross-section. (**i**) Close up on a bigger coprolite showing contents consisting of organic elements of different shapes. Bar = (**a**) 50 μm, (**b**,**c**) 200 μm, (**d**) 250 μm, (**e**–**i**) 100 μm.

**Figure 13:**
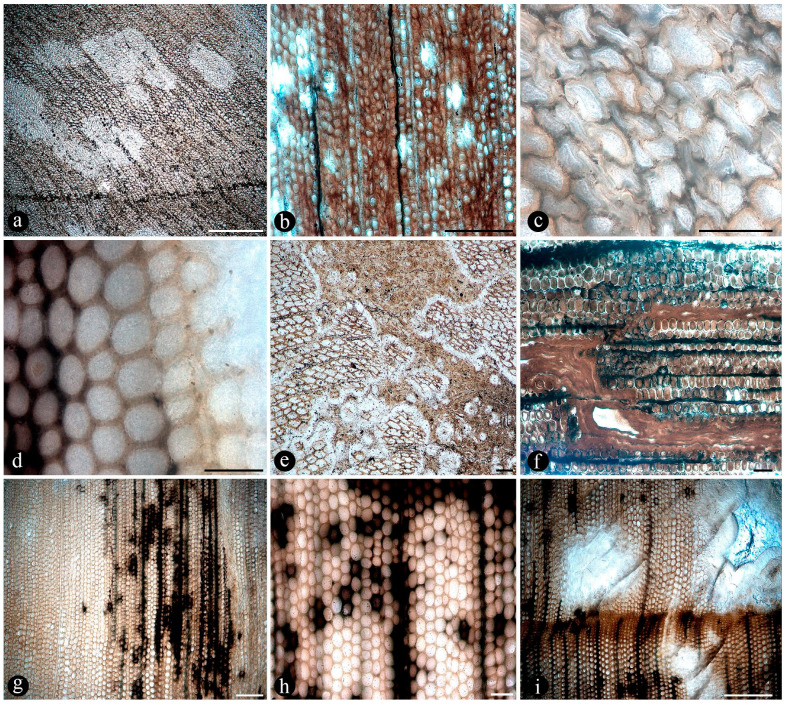
Decay patterns of associated wood. (**a**) General view of a section of decayed conifer wood with a typical mottle pattern with large, irregular decay areas. (**b**) General view of a section of decayed conifer wood with a mottle-rot pattern, characterized by numerous approximately circular decay areas distributed over sound tissues. (**c**) Close up on decay areas characterizing the mottles of the mottle-rot patterns showing deformed and hyaline xylem cells. (**d**) Close up on the margins of another mottle showing progressively decayed xylem cells towards the center of the decay area. (**e**) General view of conifer wood with large, irregular decay areas, where xylem tissues are replaced by a homogeneous and opaque mass of disrupted cells, along with cellular contents and other organic debris. (**f**) General view of a decayed area with distorted cells and massive decay spots filled with an opaque substance. (**g**) General view of wood with radially arranged decay features, consisting of regular deposits of an opaque substance along ray cells and tracheids. Note that they are set parallel and in close association with each other. (**h**) Close up on wood showing xylem cells with their lumina filled by an opaque substance. (**i**) General view of decayed conifer wood with cells with deposits of an opaque substance aligned with the growth rings. Note the decay areas around the growth rings. Bar = (**a**) 750 μm, (**b**,**g**–**i**) 500 μm, (**c**) 250 μm, (**d**–**f**) 100 μm.

**Figure 14:**
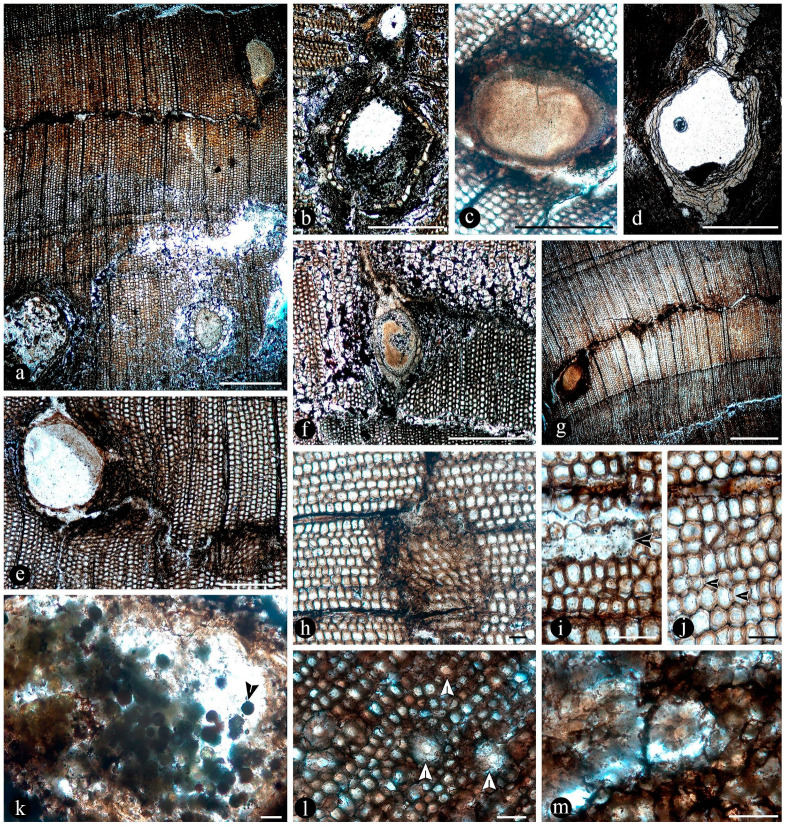
Decay patterns of associated wood. (**a**) Decayed conifer wood showing several traumatic resin ducts of different sizes aligned parallel to the growth rings and connected to each other by an opaque substance. (**b**) Close up on a diamond-shaped traumatic resin duct with a characteristic rim of hyaline subsidiary cells and abundant deposits of an opaque substance in the surrounding cells. (**c**) Close up on a smaller, elliptical traumatic resin duct embedded in an abundant opaque substance. (**d**) Bigger, irregular-to diamond-shaped traumatic resin duct surrounded by several concentrical deposits of an opaque substance. (**e**) Close up on sound xylem cells in proximity to a traumatic resin duct and an extensive deposit of an opaque substance that connects it to the growth rings (**f**) Close up an area with decayed and sound xylem cells, with a traumatic resin duct embedded within an opaque substance that connects with xylem cells aligned to the growth rings. (**g**) General view of traumatic resin ducts and parallel growth rings, connected by an opaque substance, forming a tangential barrier. (**h**) Close up around the growth rings with cells filled by opaque substances showing decayed and sound cells preserved cells around it. (**i**) Close-up on a group of xylem cells with all wall components degraded, with no tissues preserved (arrowhead). (**j**) Close up of another group of xylem cells shows some with their innermost wall component partially detached and projected to the center of their lumina. Note the intermediate cell wall components of the same cells appearing differentially decayed, with scars and pits of different sizes observed (arrowheads). (**k**) Close-up on a traumatic resin duct showing a series of globose structures trapped within an opaque substance present in its lumen (arrowhead). (**l**) Close-up on several, few-celled traumatic resin ducts, clustered or closely placed to each other (white arrowheads) within mostly sound xylem cells (**m**) Close up on some of the few-celled traumatic resin ducts showing cells radially arranged and a central lumen filled with an opaque substance. Bar = (**a**) 750 μm, (**b**–**g**) 500 μm, (**h**,**i**,**l**,**m**) 100 μm, (**j**,**k**) 50 μm.

Images of the unmounted chert blocks and their sections were obtained using a Canon (Tokyo, Japan) camera with a Canon macro lens of 60 mm (Figure 3). The same camera was used for images of extant populations of O. spectabilis (see “Comparisons” section below) (Figure 4a–e). Multiple images of the samples at several focal planes were taken to compose an image with greater depth of focus, following an image-stacking technique [37]. The images were stacked using auto-align and auto-blend functions in Adobe Photoshop 2023 (San José, CA, USA). Composite images were created using both image-stacking and image-stitching techniques to visualize the entire specimens and their surrounding matrices (e.g., Figure 3). Images of the slides were obtained with a Leica DM 2500 transmitted light microscope with an incorporated, DFC 295 Leica camera (Leica Microsystems, Wetzlar, Germany), (Figure 5, Figure 6, Figure 7, Figure 8, Figure 9, Figure 10, Figure 11, Figure 12, Figure 13 and Figure 14).

The macrospecimens and their sections are housed at the Museo Regional Padre Molina of Río Gallegos (Santa Cruz Province, Argentina) under accession numbers MPM-Pb 16084–16108.

**Surface 3D-scanning of fossils,** selected chert samples preserving one to several fern individuals were 3D-scanned in high resolution using an Artec Space Spider 3D Scanner (Senningerberg, Luxembourg). Before scanning, the chert blocks were washed with water and brushed to remove all the loose sediments. A thin coat of vegetable oil was applied to the surface to reduce brightness. The images were obtained and processed with the software Artec Studio 17, applying the mode Geometry + Texture to obtain tridimensional models of the blocks. The models exported from Artec Studio as .ply files were then converted to .u3D files using the software MeshLab 2023.12 to observe the superficial morphological features of the fern individuals, their distribution in the chert matrix, their relative position and distance to each other when more than one individual is preserved within the same block, and the distribution of other associated plant remains (e.g., conifer wood and twigs).

**For comparisons with extant Osmundaceae,** in order to make comparisons with the extant Osmundaceae species in Argentina, we studied populations of *Osmunda spectabilis* Willd. from Misiones province. The specimens were collected during field trips to the Cruce Caballero Provincial Park (−26.515012, −53.995383), herborized using traditional techniques, and deposited at the Museo Argentino de Ciencias Naturales “Bernardino Rivadavia” (BA herbarium).

In order to carry out anatomical comparisons, samples of petioles, rhizomes, and roots were fixed in a mixture of formalin, alcohol, and acetic acid [38]. Histological preparations were made free-hand and mounted using gelatin–glycerin. In some cases, methyl blue was used for staining fungal structures in order to detect fungal associations. A Leica DMLB microscope was used for observations (Figure 4f,g). The measurements related to petioles and roots presented in Table 1 were obtained from 10 samples. The measurements related to ramifications could only be taken from 2 samples, as these propagation structures were found exclusively in them.

## 3. Results

**Composition of the main chert blocks (including the spatial arrangement and habit within individual cherts and the relationships among the chert blocks)**, Osmundaceous ferns in situ preserved within 13 discrete chert blocks (up to 45 × 30 × 25 cm in size), holding up to 3 specimens together per block and around 21 individuals total occur exclusively distributed over an area of around 250 mts^2^ in the intact geothermal paleolandscape (Figure 2). Ferns are preserved embedded within the chert matrix, where mainly the rhizomatous stems, together with their persistent leaf bases and associated roots attached to the original substrate, persist in a life position (Figure 3a,b). These are accompanied by several remains of individual rhizomatous stems of osmundaceae spread over the nearby area (i.e., the majority of ~200 fossil specimens collected) and a few conifer remains, including a stump in life position with attached wood fragments, leafy branches, and seeds [25]. Within the mapped chert blocks occur from one to up to three ferns that are discernable, which vary in size (stem diameter 1.5 to 16 cm) and the angle of orientation with respect to each other (Figure 3c–e). The angle of orientation within their respective substrates is variable and includes from almost horizontal to vertical individuals. Some larger stems of 7–14 cm in diameter and 10–15 petiole cycles have smaller 1.5–4 cm in diameter subordinate individual stems with 3–6 petiole cycles attached laterally. Individual rhizomatous stems are characterized by a parenchymatous inner cortex, a clearly differentiated sclerotic outer zone, and stipulate petiole bases with a strong circular homogenous sclerotic ring surrounding the vascular bundle (Figure 3c–e). In addition, their vascular arrangement corresponds to a dictyoxylic siphonostele (Figure 3c–e). In particular, an ectophloic dictyostele with complete leaf gaps is observed, and the leaf traces separate from the xylem cylinder with only one protoxylem cluster, whose division occurs in the outer cortex (Figure 3c–e). Further characteristics include one large mass of sclerenchyma near the ring plus several aligned masses in the stipular wings of the petiole bases (Figure 3c).

Numerous dispersed sporangia, isolated or grouped, dehisced with none, a few, or completely filled with intact trilete or somewhat collapsed spores within, and also individual pinnae, were observed in the chert matrix (Figure 5a–d).

The quantitative traits used to compare the fossils with the current specimens are summarized in Table 1.

**Associated plants.** In the nearby adjacencies and beyond this main accumulation of fern-dominated chert blocks, there are also further, identical, frequently fragmentary, osmundaceous fern fossils and a greater relative number of conifers in the greater landscape [25]. These osmundaceous ferns are, in general, characterized by having a two-layered cortex, an ectophloic, dictyoxylic siphonostele with a subexarch protoxylem, C-shaped leaf traces with a circular homogeneous sclerotic ring, homogeneous parenchymatous pith, and persistent mantle of winged petiole bases and diarch adventitious roots [25].

In addition, a couple of fern rhizomatous stems hold the presence of minuscule epiphyllous bryophytes. These have not been studied in detail. Thus, their systematic affinities are yet unknown.

**Associated microorganisms**. Additionally present in the chert matrix and associated with the tissues of the ferns preserved within the main blocks, there are several microorganisms. Furthermore, the infected plant organs display a variety of decay and consumption patterns that result in modifications of the structure and appearance of the infected tissues, which sometimes also include the presence of coprolites along with several microorganisms.

In particular, in the interspaces between the fern petioles, there are several adventitious diarch roots that contain different endobiotic filamentous structures within the cortex cells (Figure 6a). The endobiotic filaments consist of hyphae that fill up some or most of the lumina of the host cells, especially of those around the pericycle (Figure 6a,b). The filaments thin-out into highly ramified structures that arise from wider trunk ones and, then, profusely ramify into multiple tiny segments that occupy most of the space of the lumina of the colonized cells (Figure 6c). Some of the host roots are filled up with fungal material and may appear obscured by a “spongy” and opaque residue, whereas in other areas of the adventitious roots, the cells are eroded or missing (Figure 6g). Included within the adventitious roots, sometimes there are also other broad coenocytic hyphae, which are also obscured by the opaque deposits within the cortex of the roots, although they can be distinguished at different focal lengths, appearing somewhat coiled and occupying most of the lumina of the host cells (Figure 6h). Accompanying the hyphae are globose structures distributed also within the host root cells (Figure 6i). The globose structures are characterized by a uni- or bi-layered wall of variable opacity and by having a short, robust, subtending hypha (Figure 6j). In addition, attached to the outer surface of some of these degraded diarch roots via a single subtending hypha, there are small globose-to-ovoid identical structures (Figure 6k).

The fossil fern stems include filaments and globose structures of different shapes and sizes distributed within the host cells or empty spaces created in the decayed tissues. In particular, adventitious diarch roots and especially petioles within the fern stems commonly appear degraded to various degrees, sometimes hollowed out and containing an uncountable number of globose, pyriform, and ameboid structures (Figure 7a,b). They vary in size (12–38 μm), wall thickness (0.5–1.5 μm), layering, shape, and arrangement (Figure 7c). The smaller forms are approximately spherical and have no contents, ornamentation, or other distinctive morphology (Figure 7c). Some of them occur as dispersed entities, individually discernable as floating units within the degraded, hollowed tissues, or they occupy the lumina of other globose structures or host cells alone in loose-to-dense groups (Figure 7c–f). Some bigger forms (up to ~54 μm) display 2–3 circular, annulate, superficial scars and/or papillae, flat, or protruding; a thicker wall that is one-, two-, or three-layered; a basal apophysis or similar apparent rhizoids attached to the substrate; and a cytoplasm or lumen that may appear granulated or somewhat divided into small subunits (Figure 7g–k). Collapsed psilate-to-faintly granulated globose structures sometimes contain smaller forms in their lumina (Figure 7l). In addition, bigger forms with clear walls cast-bowl-to-plate-like or tote-bag-like profiles, respectively (Figure 8a,b). Sometimes one to several smaller globose structures are attached to the outer surface or included in the lumina of intact and modified bigger forms (Figure 8c–e). The endobiotic forms sometimes display small protrusions that extend outwards and remain in contact with the host wall of the bigger forms (Figure 8e).

In some of the degraded petiole and root tissues, and also associated with a decayed sporangium and unidentifiable plant debris, there are inter- and intracellular filaments (~2–6 μm wide) (Figure 9a–c). Some of the filaments are wider (9–13 μm) than others and generally appear coenocytic, often completely filling the lumen of host cells (Figure 9d,e). Other filaments are more clearly septate, branched at right to acute angles, and they give rise to small globose-to-quadrangular consecutive subunits (Figure 9f–i). Other filaments have one or two branches that narrow distally and septate (Figure 9j,k). Some of these are attached to host cells by a wide circular base (Figure 9l). Other filaments present in the lumina of some of the cells within the degraded tissues are completely opaque, of smaller size, and have secondary branches with regular globose protrusions (Figure 9m).

Additionally, there are pyriform-to-elliptical, circular-to-somewhat-irregular, and flattened structures of variable size (115–70 μm) distributed within and between the degraded fossil diarch roots and petioles (Figure 10a–f). These are characterized by complex n-layered hyaline-to-opaque, psilate, reticulate, or ornamented walls (e.g., short broad spines or similar elements) and sometimes have a broad subtending hypha (Figure 10a–f). Some of these structures contain a flat, disc-like inclusion (Figure 10e–g). In addition, smaller (30–65 μm), thick-walled, opaque or hyaline, multi-celled, psilate, dispersed spores have been observed in the degraded tissues or within the lumina of host cells (Figure 10h,i). Some of these are helicoidal spores (i.e., multicellular spores with a spiraled morphology with tapered ends, and transverse septa), and others are dictyospores (i.e., multicelled spores with both transverse and longitudinal septa).

The tissues occupied by the globose structures and filaments display a range of decay features consisting of broken, torn-apart, and deformed cells, which sometimes have their lumen filled with an opaque substance (Figure 11a). At greater magnification, some cells appear to have their innermost cell wall layer detached from the remaining components and projected toward the center of their lumina (Figure 11b). Some other cells within similarly decayed areas have their middle lamellae differentially degraded, and their cell wall layers appear deformed virtually indistinguishable from each other (Figure 11c). In other areas, the cells have all their wall components degraded, remaining connected to each other mainly through the middle lamella at the corners of neighboring cells (Figure 11d). Further decay of all cell components is observed in other areas, where globose structures and filaments are present (Figure 11e,f). In other petioles, decay spots have a fuzzy or spongy somewhat smooth to-silky or velvety texture (Figure 11g). Sometimes, these decay spots or areas have a more regular shape and appear concentrated in some zones of the decayed petioles (Figure 11h). A closer look at these areas shows a radiating fusiform pattern, where the cells appear progressively decayed and, eventually, individually indistinguishable (Figure 11i–l).

In addition, degraded fossil petioles and diarch roots contain clusters of elliptical-to-cylindrical coprolites (Figure 12a–c). Fern petiole tissues display additional borings of different shapes, which are empty or filled with an amorphous organic residue and coprolites (Figure 12d,e). Present in the bored tissues are globose structures and filaments, which are attached to the degraded tissues and also to the coprolites (Figure 12d–g). The coprolites vary in size, shape, texture, and apparent contents (Figure 12f–i). Some coprolites are small (70–50 × 40–35 μm), elliptical in shape, approximately circular in cross-section, have a smooth texture consisting of finely comminuted organic particles and are distributed in the degraded vascular trace and cortical cells of fossil petioles (Figure 12f,h). Other coprolites in the degraded cortical tissues of the petioles and in diarch roots are bigger (180–140 × 80–90 μm), somewhat quadrangular to cylindrical, form loose groups of fewer individuals, and have rough textures given by some more angular contents (Figure 12g,i).

In addition to the microorganisms and decay features of the fern stems, numerous chunks of wood preserved along with the latter in adjacent chert blocks or as isolated pieces show various biodeterioration patterns. These include general areas of decay interspersed along with xylem cells that appear sound, sometimes resulting in variable mottled patterns (Figure 13a,b). Included are spots where the tissues appear hyaline or less opaque than regular xylem cells, and there are irregular-to-circular areas where the tissues are missing and completely eroded away (Figure 13a,b). In the hyaline zones, the cells are variously deformed, broken, and torn apart, whereas the xylem in the borders of the completely degraded areas shows a progressive decay, appearing increasingly decayed and hyaline inwards (Figure 13c,d). The infected fossil woods show organic residues and opaque deposits distributed in irregular-to-discrete decay areas, sometimes arranged in somewhat parallel lines that include the ray cells and regular tracheids (Figure 13e–g). In these later zones, the opaque deposits occupy the lumina of sound cells (Figure 13h). In addition, the opaque deposits sometimes are concentrated at the growth rings, around which the xylem appears partially to completely degraded (Figure 13i).

In addition, fossil wood presents several traumatic resin ducts of variable size and shape, distributed within the sound and degraded secondary xylem cells, aligned parallel to the growth rings (Figure 14a). The traumatic resin ducts are approximately ovoid-to-diamond-shaped, have an ample lumen, are empty or filled with an amorphous opaque substance, and have a ring of cylindrical-to-quadrangular bulging subsidiary cells (Figure 14b,c). Continuous with the ring of cells around the traumatic resin ducts, there are abundant deposits of an opaque substance, which may be arranged in concentric lines (Figure 14d). These deposits of an opaque substance extend into the surrounding xylem and are in direct contact with the degraded tissues and the growth rings (Figure 14e–g). Together with the growth rings, the traumatic resin ducts form a tangential line of opaque deposits, around which different decay features are present (Figure 14g,h). This includes areas with intact cells and others where the walls of the xylem cells are broken and sometimes degraded and separate into degraded individual components (Figure 14i,j). In addition, clusters of globose structures appear embedded in some of the traumatic ducts and the surrounding amorphous opaque substance (Figure 14k). In other areas with more variable decayed xylem cells, there are much smaller traumatic resin ducts that consist of a lumen the size of a single cell, which are surrounded by an incipient ring of hyaline-to-opaque line of subsidiary cells (Figure 14k,l). These smaller traumatic ducts appear clustered or close together and also display remains of an opaque substance that completely clogs their lumina, which is also spread over the surrounding area (Figure 14k,l).

## 4. Discussion

**Overview and Context**

We have described a Jurassic plant assemblage dominated by ferns of a single species of Osmundaceae, alongside some additional plant and microbiological components, which together provide a unique window on terrestrial Jurassic ecosystems. The morphology displayed by these ferns conforms with the previously described morphotaxon *Millerocaulis zamunerae* from the same locality in the adjacencies of the assemblage described here [18].

Based on the preservation in situ and in-life position of this plant assemblage within its original bearing sediments, some extrapolations regarding its composition, structure, organization, and general characteristics of its habitat are inferred and suggest that the original configuration of the assemblage became preserved. These characteristics, including the identity, habit, and distribution of the ferns and other members of the greater plant community, trophic functional characterization of microbes present in the bearing sediments and fern and conifer tissues, and the general characteristics of the paleoenvironment, are compared to modern analogous Osmundaceae-dominated swamps, such as the highland lagoons in Northeastern Argentina in the Parana Forest (Figure 4). We explore the attributes that might have made this apparently conserved ecosystem persistent through time, including those referred to its taxonomic composition based on the morphology of the preserved taxa, habit, developmental biology of the ferns, and functions and dynamics derived from the associations and interactions among its members. In turn, these characteristics are analyzed in terms of the concept of ecological stasis, which accounts for unique cases of morphological and ecological stability within flexible bounds across geological time [11,12,39,40].

**Plant community**

**Composition, development, and paleoenvironmental significance**

The fossils described here consist of around 21 rhizomatous stems, which are variable with respect to their relative size and orientation within the original bearing substrate. In addition, some examples of dispersed empty sporangia, sometimes with collapsed spores, and pinnae are also present, and these too occur embedded within the same embedding siliceous matrix. The variable shape and size displayed by the fern stocks suggest different developmental stages, whereas the presence of dispersed reproductive sexual structures is suggestive of generative and senescent life stages, which argues in favor of the preservation of the original spatial arrangement [3,41,42]. Moreover, individual ferns appear closely distributed and sometimes are bound together via lateral roots within the sediments, which represents clonal development. Osmundaceae grows vegetatively by spreading lateral, horizontal rhizomes that may break apart into individual plants within the substrate (Figure 4e), conforming to typical clonal populations. This strategy allows for rapid and effective horizontal spread within the occupied substrate, which in turn, increases the chances of biotic interactions and more efficient nutrient acquisition [43,44]. Although these are cases where slow growth rates were recorded, an age of 400 years has been calculated for populations of *Osmunda claytoniana* L. growing through clones in humid and cold forests in Western Virginia (USA) [45]. This highlights the importance of vegetative propagation for the maintenance of the species of this group, both in extant and fossil Osmundaceae communities [45].

Clonal growth organ formation in the Jurassic swamps of Patagonia can be suggested as an adaptation to the typical hydrological conditions of the geothermal setting, where oscillating water levels are related to fluctuating nutritional levels, such as nitrogen [46]. Evidence of nitrogen fixation is not known in the geothermal settings in Patagonia, although the presence of nitrogen-fixing microorganisms in acidic, modern geothermal soils supports it [47]. Moreover, clonal development is suggested to represent a reproductive strategy that regulates the frequency of sexual vs. asexual reproduction [48].

To what extent the geothermal system influenced the dynamics and structural configuration of the Osmundaceae-dominated community in the Jurassic of Patagonia may be suggested based on the relative frequency of rhizomatous stems and their mode of preservation with respect to other plants present. The majority of the fossils found are massive rootstocks, suggesting a mature community with relatively fewer rootstocks from younger life stages, akin to a senile community [41]. Within the greater paleolandscape, further rhizomatous osmundaceous stems are found in an area exceeding 2 km around, which could imply different scenarios, including the possibility that additional swamp-like settings containing analogous communities were present. However, different from the specific assemblage described here, additional rhizomatous stems generally occur as dispersed units associated with geothermal wetlands, where conifers and other ferns are also present [18,25]. In particular, the immediate surroundings of the specific area occupied by the chert blocks containing mostly rhizomatous stems also show the presence of further specimens of Osmundaceae, but as individual floating units, in a proportion of approximately 9 to 10 compared to conifer remains, the other plant group observed. The combination of anatomical features exhibited by these rhizomatous stems places them within the Osmundoideae subfamily and most likely also corresponds to *M. zamunerae* [18,25].

This extensive distribution of the Osmundaceae in the paleolandscape suggests that the geothermal system influenced its organization and structure. In turn, the dynamics of the system and the typical mode of preservation within geothermal systems, where everything below the water table level in the swamp setting likely had equal chances for preservation, appears as an ideal setting preserving a reliable window of the original composition and organization of the Osmundaceae-dominated assemblage in the Jurassic (Channing, 2017). In this, in situ preservation of the Osmundaceae community within its original substrate, closely replicating its original structural configuration, seems possible. Compositionally, the assemblage is dominated by a single species. But, a few conifer remains are also found in the main chert blocks, whereas in the greater landscape, additional fern and conifer fossils are present. The relative absence of trees within the nucleus of the osmundaceous paleocommunity is noteworthy and consistent with modern assemblages, where open environments lacking shade-producing plants are positively correlated with the development of generative life cycle stages and overall healthy populations of *Osmunda regalis* L.-producing sexual structures [41,42]. The absence of trees within the core of the Osmundaceae-dominated community suggests that they were not adapted to the swampy conditions containing the ferns.

In these restricted settings of the Jurassic of Patagonia, several individuals of osmundaceous ferns were distributed in an exclusive area of ~250 m^2^ in monotypic stands and were preserved in life position, where conifers, other plants, or their remains are absent or only sporadically observed. However, although within the core of the Osmunda-dominated assemblage, there are almost no other plants preserved, except for some dispersed Osmundaceae rhizomatous stems (i.e., not forming part of the main blocks containing the insitu and in-life position rhizomatous stocks) and fewer conifer organs (e.g., foliose branches, wood), he possibility of additional plants present cannot be discarded. Therefore, it remains possible that additional plants in the same area or in the greater landscape did not undergo preservation in the same manner as the fern rhizomatous stems. These would include additional Osmundaceae, along with an undetermined number of conifer species of the Araucariales Gorozh. Ref. [25] shows that herbaceous ferns, comparable to the Gleicheniaceae, Dicksoniaceae, Cyatheaceae, and Equisetaceae Michx. ex DC were also widespread across an area of ca. 4 km^2^ in the greater paleo-geothermal settings of the La Bajada locality in Patagonia [18,25]. This suggests that a broader combination of plants contributed to the composition and dynamics of the Osmundaceae-dominated assemblage.

Conifers are present in both Jurassic and modern settings, accompanying the Osmundaceae in the greater landscape, but their taxonomic affinities and relative abundances coincide only partly at the family level (i.e., in both fossil and modern Osmundaceae-dominated settings, Araucariales are present). Despite logical taxonomic shifts in species-specific compositions, the main similarities between the Jurassic and the modern settings regarding the distribution, relative abundance, and species-specific dominance of Osmundaceae appear as conserved features. Moreover, fossil preservation in chert blocks containing the in situ rooted osmundaceous ferns within the original bearing swampy sediments suggests reduced time-averaging differences with respect to the original assemblage. Therefore, a good chance for a significant idea of the original composition and structure of the Osmundaceae community is possible.

This record shows that the taxonomic composition of the Jurassic Patagonian assemblage, compared to modern settings, has varied and that the distribution of Osmundaceae-dominated communities in the swamp paleosettings has an analogous modern form of restricted geographical distribution. Moreover, whereas ferns and conifers made up the main plant groups during the Jurassic, modern ecosystems have a greater number of components, particularly angiosperms, which were absent during the time of formation of the fossil assemblage in Patagonia [5]. Also, at an even greater scale, the Jurassic of Gondwana was dominated by conifers (i.e., Cupressaceae Gray, Araucariaceae Henkel and W.Hochst., Podocarpaceae Endl., and Cheirolepidiaceaee Hirmer et Hörhammer [49,50], which formed forests of variable density, where herbaceous and shrubby vegetation belonging to different fern groups (e.g., Gleichenaceae, Dipteridaceae Seward and E.Dale) and other gymnosperm groups (e.g., Cycadales Pers. ex Bercht. and J. Presl and Bennettitales Engler) thrived in understory, more or less open, settings [49,50].

**Most similar modern analogous settings**

Modern high-elevation open swamps in Misiones, Argentina appear as the most closely comparable analogs to the Osmundaceae-dominated communities in the Jurassic geothermal landscapes of the Deseado Massif in Patagonia. As in the modern swamps, the paleo-settings in Patagonia were dominated by monotypic stands of Osmundaceae, accompanied by ferns of other families and conifers. Evidence shows that these accompanying plants were the second most important ecological groups of the ecosystem, considering their relative abundance and distribution in the paleo-geothermal landscape. This characteristic organization of the Osmundaceae-dominated landscape of Patagonia was probably influenced by the dynamics of the migrating geothermal system. This effect may be analogous to the influence that the frequent inundation caused by small-scale fluvial systems in the Paraná Forest highlands has over the development of modern Osmundaceae-dominated fern ecosystems in Misiones [51].

The wetlands or swamps of the Paraná Forest in Northeastern Argentina are small depressions that form more or less open vegetated lagoons at relatively high elevations (~500–800 m.a.s.l.) on gently sloping land or paleochannels, which are generally located in the forest and fed by creeks of variable magnitude [51]. They are also characterized by their organic-rich substrates, which are correlated to high species richness and endemism. In these kinds of habitats, Osmundaceous ferns constitute persistent hydrophilous communities (Figure 4a,b) together with marsh plants and shrubs that are surrounded by trees, including the conifers of Araucariaceae (*Araucaria angustifolia* (Bertol.) Kuntze). These swamp settings are variable with respect to their more specific composition, and some of them are formed by monotypic associations of osmundaceous ferns or associations of Osmundaceae with other ferns with a similar habit, such as *Neoblechnum brasiliense* (Desv.) Gasper and V.A.O. Dittrich (Blechnaceae) and *Cyathea atrovirens* (Langsd. and Fisch.) (Cyatheaceae) (AY observation). Some key environmental parameters of these settings include acidic soil pH, water availability, reduced tree cover, and stable environmental conditions.

Some features characterizing the Jurassic Patagonian assemblages appear to mimic the modern conditions that are typical in the Paraná Forest’s highland lagoons, including a low sedimentation rate, organic-rich substrates, and undulating paleotopography. The pH of the osmundaceous environment may have varied from alkaline to neutral to slightly acidic in response to the dynamics of the migrating thermal waters, being alkaline to neutral during periods of inundation and acidic during dryer phases. While no direct pH measurements have been obtained for this setting, the typical textures of the studied fossiliferous cherts are consistent with this hydrological regime. Similar variations in the pH regime, frequency of sedimentation, and abundance of organic-rich sediments were inferred from chert textures and other analyses in different geothermal localities in the Deseado Massif and described for distal environments in general [31,52,53,54]. 

In addition, the preserved anatomical characteristics of the fossil Osmundaceae rhizomatous stems, which conform to the previously described morphospecies *Millerocaulis zamunerae*, are also generally like those of *O. spectabilis* which are presently adapted to wetlands, swamps, and similar environments [18,51] (Figure 4d,e). The similarities include the size and disposition of petioles and roots (Table 1), as well as the arrangement of sclerotic tissues, which form a continuous layer around them [18]. Such traits indicate a functional and ecological parallel between the fossil and modern osmundaceous communities.

In addition, Osmundaceae as a family is generally restricted to mild climates and wet biomes, but the distribution of morphotaxa within species varies with elevation, mean annual temperature, temperature seasonality, and annual precipitation, which in time, is reflected in their morphological traits [13]. For instance, the morphotaxon ‘palustris’ of *O. spectabilis* characterizes the high-elevation swamps with high mean annual temperature and precipitation in South America, including Northern Argentina. Similarly, stunted forms of *O. regalis* are typical of high-elevation swamps in Africa and regional islands, suggesting possible genetic connections [13]. There is no direct measure of what the possible paleoelevation of the Patagonian geothermal settings was when the Osmundaceae monotypic assemblages were alive, but the abundance of araucarialean remains is suggestive of a hilly landscape. Additionally, field geological evidence indicates that the osmundaceous communities occupied relatively depressed areas within the greater landscape, which is akin to swamp-like settings [22,46]. Alternatively, comparisons in an environmental and climatic framework between the fossils described and the current *Osmunda* L. populations are difficult to establish because the morphological characters used in extant analyses [13] are predominantly related to external morphology, principally of the fronds. Additional comparative anatomical studies within the genus are necessary to contribute to the discussion on paleobotanical assignments and the relationship of taxa to the environment.

Therefore, while the Jurassic Osmundaceae communities from Patagonia share structural and environmental characteristics with modern Osmundaceae-dominated swamps, the apparent taxonomic differences reflect that ecological and evolutionary shifts occurred over millions of years. Notwithstanding this, the Osmundaceae-dominated community is a unique reference about the organization and dynamics of typical fern communities in the Jurassic of Patagonia, and serves as a point of reference to understand the long-term adaptability and persistence of Osmundaceae in swamp-like environments.

**Microbial community**

**Composition, affinities, paleoecological significance**

**Mutualism and glomeromycete fungi**

Evidence that members of the Osmundaceae paleocommunity engaged in mutualistic associations with glomeromycete fungi is based on the presence of abundant fossil adventitious diarch roots of variable size with numerous typical structures interpreted as intracellular arbuscules and spores (Figure 6a,b,g–k) [55,56,57]. The presence of some fossil diarch roots with arbuscules with different preservations is consistent with the active formation of endomycorrhizae in the Jurassic Paleoecosystem [58,59]. Moreover, the distribution of arbuscules is identical in the fossil and modern Osmundaceae, such as in *Osmunda spectabilis* from swamps in the Paraná Forest highlands in Northeastern Argentina (Figure 4f and Figure 6a–f). Other alternatives indicated by the arbuscules with different preservations, such as non-functional or transient mycorrhizal associations, and saprotrophic or parasitic interactions are also possible [58,59,60]. The status of the potential interactions, in which the fungi and the individual osmundaceous ferns engaged based on the observed association, may have been influenced by different aspects of the interacting biotic communities and the environmental conditions [61,62].

The development of endomycorrhizae is influenced by environmental conditions, and the effects of their formation may be expressed at a later stage of development, such as in clonal offshoots from mother plants, having a wider ecological impact at the community level and, in turn, in ecosystem functioning [63,64,65,66]. Then, the formation of endomycorrhizae by the Jurassic osmundaceous community would have been a valuable adaptation to the dynamics of the geothermal system, such as via increased resource acquisition, allocation, biomass growth; clone formation promotion; and areal expansion [52,65,67]. Moreover, as it has been shown that a preferential formation of endomycorrhizae is less likely in waterlogged soils and aquatic settings in general, it can be suggested that the osmundaceous community likely occupied sufficiently drained soils where, this type of interaction was possible [61,68]. Indeed, modern examples of endomycorrhizae illustrated in this paper, formed by *Osmunda spectabilis* in the highland lagoons in the Paraná Forest, correspond to small adventitious roots present normally at the water–air interphase and above (Figure 6d–f).

Endomycorrhizae associations enhance nutrient acquisition, stress tolerance, and most metabolic functions in modern settings, especially in unstable ones, like the Jurassic geothermal ones from the Deseado Massif in Patagonia and similar modern swamps inhabited by Osmundaceae [61,69,70]. The conditions that are typical of the geothermal system may have influenced endomycorrhizae formation while gradually entombing and preserving a unique window into Jurassic Osmundaceous landscapes in Patagonia. Endomycorrhizae formation could have been facultative, readily developing to cope with the periodically disturbing input of the geothermal system [71]. Despite whether they were facultative or always active, formation of endomycorrhizae and clonal growth organs by the Osmundaceous fern community is a remarkable feature of the Jurassic swamp settings in Patagonia. The formation of endomycorrhizae in the osmundaceous community provides a point of reference for the structure and dynamics of this paleoecosystem, highlighting another analogy with osmundaceae-dominated modern swamps. In turn, this record shows that this type of mutualistic interaction has been an important component of the trophic structure of Osmundaceae-dominated systems in general, suggesting a crucial role in ecological community stability at least for the last 150–170 Ma (Middle-Late Jurassic).

**Saprotrophy and parasitism**

**Protists and Fungi**

Numerous masses of morphologically variable globose structures, typical in the decayed organs of Osmundaceae morphologically resemble the thalli of different protists and fungi, including some testate amoeba, algae, peronosporomycetes, protozoa, and chytridiomycetes and dispersed mitospores (Figure 7 and Figure 8) [72,73,74,75,76,77].

Based on shape, specific morphological features, habit, and size (Figure 7c–l and Figure 8a–e), they can be related to different unicellular algae (e.g., *Chlamydomonas* Ehrenb; *Volvox* L.); *Chlorella* Chlorella, *Chlorococcum* (Schrank) Meneghini, *Desmococcus* F. Brand, and *Pleurococcus* Meneghini), which can be found in similar modern settings [75]. Some of the fossils from Patagonia also look like some protists from modern freshwater settings based on their size and shape, including testate and naked amoeboid forms such as *Arcella* Ehrenberg, *Pelomyxa* Greef, *Nassula* Ehrenberg, and *Prorodon* Ehrenberg, *Mayorella* Schaeffer [75,77]. Other forms appear sectioned and display certain unique morphologies (e.g., a granulated appearance and papillae, rhizoids, apophyses) (Figure 7g–k) that morphologically resemble testate amoeba and ciliate protozoa [78,79,80,81]. In addition, some of the endobiotic globose structures look like the cystosori of plant-parasitic Plasmodiophorales Karling (e.g., *Membranosorus* Ostenfeld and H.E.Petersen, *Anisomyxa* B.Nemec) [82]. Even other big globose structures filled with smaller units (Figure 8b–e) are reminiscent of peronosporomycete (water molds filamentous protists) oogonia, such as *Pythium* Pringsheim and *Achlya* Nees [78,83,84]. Fern host cells filled with small globose structures (Figure 7f) also look like endobiotic oogonia, such as *Olpidium* (A.Braun) J.Schröt., *Rozzela* Cornu [83]. Based on shape and size, the globose structures are also reminiscent of dispersed extant and fossil mitosporic fungal spores, although they differ in having more complex apertures, attachment structures, and habits [73,84].

Another group to which the fossil globose-to-pyriform structures can be related, based on shape, size, habit, habitat, and specific morphological structures, are the Chytridiomycetes [74,85,86,87]. There are several zoosporangias of extant chytrids that are most similar morphologically to the fossils from Patagonia, such as different *Rhizophydium* Schenk and *Chytriomyces* Karling [74,86]. Some of these features, including the papillae, rhizoids, and various scars or apertures (Figure 7a,b,g–j), are reminiscent of the morphologies of pre-dehisced and dehisced *Rhizophydium* zoosporangia, such as *R. megarrhizum* Sparrow, *Rhizophydium vaucherii* Wild., *R. patellarium* Erh.Scholz [74]. Moreover, some of the fossil globose structures show their lumina granulated (Figure 7k), which also looks like developmental stages of *Rhizophydium*, *Chytriomyces*, and other parasitic chytrids [74,86,87]. Other putative endobobiotic zoosporangia display discharge-like tubes hosted in the lumenina of bigger forms, and these also look like the developmental stages of other chytrids, such as *Rhizophydium globosum* and *R. sphaeroteca* [74,86]. Some sections of the infected fossil petioles show areas with cells filled with putative endobiotic zoosporangia, which are reminiscent of different chytridiomycetes with eucarpic zoosporangia (Figure 7f). In addition, a few of the globose structures have additional wall layers (Figure 7i,k) that appear more robust and opaque, which are reminiscent of the resting spores produced by the same and other extant chytrids [74,86].

Several of the putative fossil zoosporangia show features (Figure 7 and Figure 8) that are consistent with different developmental states, which also supports affinities to different extant forms and an active chytrid community [74,86,87,88]. Chytrids have been found to be the main decomposers of suspended particulate organic matter in freshwater settings, such as in swamps where different plants, including osmundaceous ferns, are present [89]. This conspicuous community of chytridiomycetes probably engaged in critical saprotrophic and parasitic interactions in the Jurassic ecosystem and contributed to the recycling of disseminated organic matter in the Osmundaceae-dominated swamp setting [89]. In addition, the observed abundance of chytrids is consistent with acidic pHs and low salinity and, in turn, with available organic remains and aerated substrates, suggesting shallow freshwater wetlands or similar settings [90].

The fossil chytridiomycetes are found in decay areas of the osmundaceous rhizomatous stems, which possibly provide a protected and stable environment [91,92,93]. The primary role of chytridiomycetes as the main organic decomposers in swamp settings aligns well with their distribution in the decayed and hollowed Osmundaceae fern rhizomatous stems [94]. Previous reports of chytridiomycetes paleocommunities in similar hollowed-out fern stems with root mantles support their ecological importance in organic matter breakdown, especially of fern tissues and other trapped plant litter, which could have aided ferns in nutrient acquisition [94]. This supports the importance of fern rhizomatous stems as particular ecological microniches sustaining diverse communities of meso- and microorganisms, which in turn, influenced the trophic structure and dynamics in the swamp setting.

The recurrent association of saprotrophic and parasitic organisms with decaying fern rhizomatous stems underscores the stability and resiliency of this microecological niche across geological times. It provides an idea of the abundance, morphological diversity, biological affinities, and roles carried out by chytridiomycetes in ancient terrestrial settings, such as in the swamp settings of the Jurassic of Patagonia. This underscores the presence of additional functional groups and provides a point of reference for the structure and dynamics in the Jurassic Osmundaceae-dominated swamps of Patagonia. In turn, possible comparisons between fossil and modern swamps dominated by Osmundaceae are faced with limitations due to differences in the actual microbial diversity documented. Despite apparently different taxonomic compositions, the fungi (and additional microorganism groups present) were at some point functional to the equilibrium and overall dynamics of the system dominated by the Osmundaceae. Moreover, this record supports the critical role of chytrydiomycete-driven recycling of organic matter in maintaining the trophic dynamics within the Jurassic community dominated by Osmundaceae.

In addition, a morphologically diverse association of filaments associated with the degraded fossil rhizomatous stems is consistent with saprotrophic activity and other interactions. Based on size, ornamentation, shape, extension, septation, branching, and opacity, they can be compared to the vegetative and reproductive structures of different mitosporic fungi [74,95,96]. Some forms produce phialide-like conidiophores (Figure 9j,k), like those of opportunistic plant-parasitic and saprotrophic ascomycete anamorphs, such as different *Penicillium* Link and *Trichoderma* Persoon species [97,98,99,100]. Moreover, some of the filaments bearing phialide-like conidiophores are firmly attached to host cells (Figure 9l) by a broad structure that looks like an appressoria, such as in some parasitic *Penicillium* [101]. Additional septate filaments produce terminal arthrospores (Figure 9g,i), which are produced by mitosporic fungi under stressful conditions [102]. The comparatively smaller, opaque, and branched filaments present (Figure 9m) are morphologically comparable to conidiospores produced in aerial hyphae by some filamentous bacteria, such as *Streptomyces* Waksman and Henrici, a widespread plant saprotroph [103]. Other coenocytic filaments (Figure 9e,f) present profusely fill the lumina of host cells in a contorted fashion and are reminiscent of those produced by some endomycorrhizal fungi within host cells [104].

Additionally, widespread globose-to-flat spores of circular-to-somewhat-irregular outlines display variable habits, shapes, walls, and ornamentations (Figure 10a–g) that look like those present in filamentous protists and algae [75,78]. Some of the encysted structures have a short extended neck or subtending hypha and at least four wall layers (Figure 10b–e), which are comparable to different peronosporomycete oogonia [75,78]. Moreover, an apparent central oospore (Figure 10e–g) is sometimes present, further supporting their similarity to peronosporomycete oogonia [78]. In addition, some of them are reminiscent of previously found spiny oogonia of the Combresomycetales (Figure 10f,g), which were suggested as saprotrophs and parasites in similar paleoenvironments [5]. Others disc-like structures have trapezoidal, individual ornaments (Figure 10d), which look like prasynophycean algae resting spores [75]. Some multicelled structures (Figure 10h,i) that are also present look like conidia or sclerotia produced by some anamorphic fungi, including some *Helicomyces* and similar helicoidal forms, which are found in freshwater settings as saprotrophs. Others look like typical saprotrophic and parasitic mitosporic fungi, such as *Hermatomyces* Spegazzini [74,95,96].

This consortium of fungi and fungi-like propagules provides additional evidence of the saprotrophs and parasites that contributed to the recycling of organic matter within the Osmundaceous community. Their presence provides additional evidence of the trophic complexity of the microhabitat represented by the rhizomatous stems and their bearing sediments.

The formation of clonal growth organs, such as in Osmundaceae, promotes development and spread via colonization, which influences mutualistic and saprotrophic fungi [105]. This reproductive strategy increases the net production of available host organs for mutualistic and organic matter for saprotrophic fungi and increases the area of distribution of these associations in the ecosystem [66]. This relationship between the Osmundaceae and mutualists and saprotrophs is evidence of another trophic mechanism connecting the ferns and microorganisms present in the Jurassic ecosystem, which probably feedback on each other, adding stability to the structure of this paleocommunity, replicating the dynamics of modern swamp settings [66,106].

**Decayed and bored fern and conifer woody organs**

Some of the fern tissues display several decay features, which were probably caused by the fungi and additional microorganisms and mesoorganisms present. Decayed fern organs display torn-apart, broken, and deformed cells, sometimes with their lumina filled with opaque substances (Figure 11a–c), which can be compared to fungal and bacterial decomposition of wood [107].

Some decayed areas appear bleached, possibly because the lignin-rich cell components are degraded, which is consistent with white-rot fungal decay (Figure 11a) [107]. Some cells have only the S2 and S3 cell wall components preserved and sometimes remain loosely attached by the middle-lamella, as when most lignin-rich components have been differentially decayed (Figure 11d,e), such as in white-rot fungal decay [107]. White-rot decay is a unique strategy deployed by some fungi that preferentially degrades lignin-rich or all cell components, including the primary and secondary walls, minimizing competition for woody resources with other decomposers [107].

Decay features sometimes appear concentrated in some areas, together with filaments and globose structures, or alone, and containing variously decayed cells, sometimes with their lumina filled by opaque contents (Figure 11a), which look like metabolic remains from natural decay processes or induced by different kinds of interactions [108,109,110]. The resulting appearance is spongy, with textures varying from smooth to somewhat velvety or silky (Figure 11j–l). These textures look like those in wood decayed by erosion bacteria, which drive wood decomposition in waterlogged settings [111]. Moreover, at greater magnifications, degraded tissues show cells with thinned walls, sometimes from the lumen outwards, and others completely dissolved, or deformed and collapsed, having their primary wall and middle lamella partly broken or entirely degraded and separated from each other (Figure 11c–f). These features can be related to decay by soft-rot fungi and erosion bacteria [111]. Other features are consistent with soft-rot decay patterns, such as cells having some components of the secondary wall (S2–S3) separated from each other and crumbled inside their lumina (Figure 11b,c) [107]. In turn, this indicates that the decay of large fern organs in the Jurassic ecosystem occurred largely in waterlogged and low oxygen conditions [112].

The patterns observed are consistent with an active and diverse community of saprotrophs adapted to various microenvironmental conditions that, through different strategies, transformed the structure of the rhizomatous stem [88,89]. Within this, plant-degrading bacteria and fungi likely influenced each other and the overall organic matter breakdown, especially over decaying fern organs. These processes show that additional functional groups of fungal and bacterial affinities contributed to the structure, dynamics, and equilibrium of the ecosystem dominated by the Osmundaceae.

In addition to the possible protists, fungi, and bacteria associated with the fossil rhizomatous stems, evidence of mesoorganisms is based on the presence of degraded and bored petioles and diarch roots, hollowed by irregular borings, some of which are filled with coprolites of two different kinds (Figure 12a–c). Smaller coprolites (Figure 12a,f,h) are related to oribatid mites based on shape, cross-section, texture, fabric, and distribution within irregularly bored fern tissues [113,114,115]. Oribatid mites are typical scavenging and opportunistic micro-detritivores that irregularly bore and feed on live and dead plant organs and debris, including fern rhizomes, which were widespread in Paleozoic and Mesozoic terrestrial ecosystems [116,117]. Additional coprolites present are comparatively bigger and have different shapes, textures, fabrics, and sizes (Figure 12c,g,i), based on which affinities to coleoptera are suggested and, perhaps, to curculionids (Curculionidae), based on the shape and contents [118,119,120]. Bored fern roots and petioles show signs of fungal decay, suggesting a correlation with fungal activity [121]. This has been previously indicated in modern and fossil ecosystems, where wood-inhabiting coleopterans and fungal decomposers mutually enhance wood decay processes [122]. This is advantageous for the interacting partners; fungal decay activity preconditions host woods for the saproxylic coleopterans, and in turn, the fungi are dispersed phoretically among potential hosts [123]. Shape-wise, the bigger coprolites can also be compared to the feces of some plant-consuming suspension-feeder aquatic invertebrates [124].

In addition, embedded in the chert blocks or isolated within the area of the osmundaceous community, there are some chunks of wood that show various features that are relatable to decay by fungi. Included are woods showing various mottled patterns consisting of irregular completely or partially degraded areas of secondary xylem interspersed within sound tissues (Figure 13a), which are typical of decay by white-rot fungi [107]. Xylem cells in these appear hyaline, deformed, broken, and torn apart (Figure 13c), possibly because of the selective decay of opaque lignin-rich components and the consequent loss of rigidity that is typical of white-rot fungal decay [107]. Other decayed, irregular, and circular-to-ovoid areas show no tissues preserved (Figure 13b,i), which is also consistent with white-rot fungal decay of all cell wall components [107]. Sometimes, decay is more discretely distributed over ray cells (Figure 13f–h), which coincides with the decay strategies followed by white-rot fungi degrading the wood of extant trees [107]. Decayed fossil wood with residual xylem cells embedded together with an opaque substance in these areas (Figure 13e,f) look like necrotic zones, which include deposits of secondary phenolic resin products used as a plant defense mechanism [108,109,125]. These putative ergastic deposits are sometimes more regularly oriented, following the axial, tangential, and parallel orientation of growth rings (Figure 13g–i), perhaps coinciding with latewood thick-walled fibers or the discrete lines in intercellular spaces between degraded xylem cells, which are reminiscent of the structured response to fungal invasion deployed by some modern trees, such as a reaction zone [110,126].

The additional woods observed show numerous axial traumatic resin ducts in the secondary xylem (Figure 14a,g), which are commonly produced by some conifers, including Pinaceae, Taxodiaceae, and Cupressaceae, constitutively in response to different types of stimuli, such as from pathogenic microorganisms and arthropods, as well as from abiotic ones [127,128,129,130]. The traumatic resin ducts in the fossil woods are tangentially aligned with respect to the growth rings, forming discontinuous rows, and occur in tissues with signs of white-rot fungal decay (Figure 14a,f,g) which could have been the causal agents [131]. Indeed, abundant deposits of an opaque substance connect the traumatic resin ducts and tracheids aligned with the growth rings (Figure 14f–h), forming one or more continuous tangential barriers that mimic similar compartmentalization strategies deployed by modern trees against invading agents [126,132]. These physical barriers, connecting directly to the traumatic resin ducts around the decay areas, appear discretely distributed (Figure 14a,f,g), suggesting an active defense strategy [126,132].

Within the degraded xylem, examples of variously deformed and broken cells, sometimes showing differential and selective decay by white-rot fungi, are present (Figure 14i) [107]. Other xylem cells have signs of soft-rot decay, such as preferential decay of the S2 secondary cell wall layer (Figure 14j) [107]. Among the degraded xylem cells, also present are smaller incipient traumatic resin ducts and rays filled with additional opaque material (Figure 14h,i,m), which supports a dynamic response to pathogens, such as some fungi [107,110,133]. These smaller and fewer-celled ducts are consistent with metabolically active tissues near the cambial zone, which likely formed in response to ongoing external stimuli, involving both chemical and anatomical adaptations [126,129]. Moreover, globose structures of unknown affinity trapped inside some of the traumatic resin ducts may be showing an additional example of the use of the barrier generated by the massive extrusion of resin (Figure 14k) [131].

These fungally-attacked woods provide additional evidence of the complex network of interactions that characterized the osmundaceae-dominated swamps, where fungi and other microorganisms played significant roles in nutrient cycling. This provides evidence of other functional groups present and of the structure of the Osmundaceae-dominated community.

The microbiota associated with the decayed woods and especially with the sheathed rhizomatous stems of Osmundaceae underscores their role as ecological microniches in the Jurassic swamp settings of Patagonia, highlighting numerous interactions between different fungi, bacteria, and protists and members of the plant community. These diverse interactions are specific to nonspecific across the different interacting organisms and replicate those in modern Osmundaceae-dominated communities and swamps and similar settings, including other fern and conifer communities, where they play important ecological roles [134,135,136,137,138,139]. The different examples observed within the Osmundaceous community provide a point of reference about their origin and historical ecological significance and new information on the biotic interactions between microorganisms, especially fungi and osmundaceous ferns in the geological record.

The documentation of diverse saprotrophs, epiphytes, and detritivores in the rhizomatous stems of fossil and extant Osmundaceae is consistent with the historical persistence of this ecological microniche, hosting a stable community across geological time [137,138,140]. The complexity added to the trophic structure of the ecosystem probably provided a buffer against ecological disturbances, such as from volcanism and associated geothermal activity, and maintained its stability through functional redundancy within the ecological groups present. This unique glimpse of the interactions in the ecosystem offers evidence of its structure and dynamics in and around the Osmundaceae rhizomatous stems, hosting a consistent array of functional groups despite possible historical shifts in taxonomic composition, which enhanced the stability of the ecosystem. A clearer picture of the ecosystem would result from the study of the microbiological community beyond the rhizomatous stems and wood, where additional functional groups present would have also greatly contributed to the structure and dynamics of the Osmundaceae-dominated community.

## 5. Concluding Remarks

The analyzed Osmundaceae community from the Jurassic of Patagonia appears as a relictual type of analogous modern communityies, in which evolution preserved its characteristic ecological organization. In turn, this suggests that its apparently complex level of organization was a constraint over the effects of disturbance from lower hierarchical ones, resulting in greater stability due to the gained resiliency of the system [40].

The characteristic ecological organization, composition, and paleoenvironment of the Osmundaceae-dominated swamp settings are most similar to some modern ones, especially those developed in the highlands of the Parana Forest in Northeastern Argentina [51]. These characteristics, including restricted sedimentation ratios, constant water availability, neutral-to-acidic soils, abundant sun insolation due to the lack of canopy vegetation, and the absence of frequent major-scale ecological disturbances, are characteristic of Osmundaceae-dominated communities in modern swamp settings [51]. In addition, some more specific features of the fossils that were found, such as the conserved morphology of the osmundaceous ferns with typical adaptations for swamp settings and the types of interactions detected, including different functional groups, add other important attributes to the system, which contributed to the persistence and stability of the system across geological time.

The recurrent association of Osmundaceae to these characteristic swamp, along with other functional biotic groups and the complex network of interactions developed, are attributes that are consistent with a stronger basis for avoiding disrupting evolutionary change in multiple lineages, adding stability to the osmundaceous fern-dominated community over time [12,20,40]. Further factors, including historical, biogeographical, population size (law of large numbers), niche differentiation, and biotic interactions within the Osmundaceae-dominated Jurassic swamps from Patagonia, might have also added other constraints that pre-empted structural changes.

However, although at the ecological scale the ecosystem remained fully functional and structurally analogous through time, its composition at a lower selecting level (species) was subject to selection because disrupting events also likely occurred [12,13,51]. This suggests that, at the macroevolutionary level, the paleoecosystem dominated by the Osmundaceae remained fully functional, maintaining its structure and energy flux, containing fossils belonging to the same macro-taxonomic groups, but of different specific compositions [141,142]. This is consistent with a dynamic equilibrium where the exchange and movement of matter within the ecosystem likely fluctuated within some ecological bounds, although without modifying its main structure [12].

In turn, this study shows how well-preserved whole plant communities in life position and in situ within their original substrates, rather than isolated organisms, enable detailed paleoecological reconstructions [3]. Moreover, the study of this paleocommunity provides evidence that shows that, despite the range of disturbances that some communities can experience across time, their persistence in analogous configurations is possible. However, additional data providing a more continuous record of Osmundaceae-dominated communities and more detailed information about the functional groups present, allowing a deeper look into the trophic composition and dynamics of the paleoecosystem, would favor a better understanding of this case with respect to the concept of ecological stasis.

## Figures and Tables

**Table 1:** Comparative measurements of the rhizome between fossil samples studied in this article and current specimens of *Osmunda spectabilis* from Misiones (Argentina). Lack of measurement of certain features in some specimens is related with the preservation (e.g., fragmentation or sectioning, orientation of the specimen, incompleteness, deformation), or it is not applicable (e.g., there are no ramifications).

Samples		Distance Between Individuals from Center to Center (cm)	Diameter (cm)	Number of Petiole Cycles	Ramifications Measurements (Smaller Individuals)			Petioles Diameter (cm)	Roots Diameter (cm)
					Ramification	Diameter	Number of Petiole Cycles		
MPM-Pb 16096	OSLB-08 Big block 1	15	7	7	-	-	-	-	-
			12	9	-	-	-	-	-
MPM-Pb 16097	OSLB-08 Big block 2	20	13	15	1	3	5	-	-
		-	-	-	2	3	5	-	-
		-	-	-	3	4	6	-	-
		-	7	10	1	-	-	-	-
		-	6	8	1	-	-	-	-
MPM-Pb 16086	OSLB-06-2	-	5	5	-	-	-	1.5–10	0.6–1.8
MPM-Pb 16087	OSLB-06-6	-	13	10	1	-	-	-	-
MPM-Pb 16088	OSLB-06-8		10	10	-	-	-	1.8–73	-
MPM-Pb 16089	OSLB-08-6	-	2.5	5	-	-	-	-	-
		-	1.5	3	-	-	-	-	-
MPM-Pb 16090	OSLB-07-2	-	8	9	-	-	-	-	-
MPM-Pb 16091	OSLB-10-4	-	14	13	-	-	-	-	-
MPM-Pb-16084-85	OSLB-01 Big Block		14	10					
MPM-Pb 16092	OSLB-06-12	-	-	-	1	1.5	3	-	-
MPM-Pb 16093	OSLB-07-7	-	16	12	-	-	-	-	-
MPM-Pb 16094	OSLB-07-6	-	11	10	-	-	-	-	-
MPM-Pb 16095	OSLB-06-7	-	12	9	-	-	-	-	-
Yañez et al. 596		6.2	4.6–7	5–7	1	1.2	2	4–10.2	0.35–3

Collected vouchers. ARGENTINA. Misiones: Dto. San Pedro, Parque Provincial Cruce Caballero, −26.515012 −53.995383, 22-V-2022, Yañez et al. 596 (BA).

## Data Availability

The original 3D-scanning files presented in the study as part of Supplementary Material are openly available in FigShare (https://doi.org/10.6084/m9.figshare.27868176.v1). In Argentina, fossils and fossil localities are considered part of the Cultural Heritage. Thus, they are protected by law (Ley Nacional 25 743, Ley de Protección del Patrimonio Arqueológico y Paleontológico). Additional legislation protects the fossils and fossil localities of Santa Cruz Province (Ley Provincial N.2472, Ley de Protección del Patrimonio Cultural). Thus, the GPS coordinates are associated with the specimens housed at the Museo Regional Padre Molina of Río Gallegos (Santa Cruz Province, Argentina) and are available upon request to the Culture Bureau of Santa Cruz Province. See Section 2 for the accession numbers.

## Supplementary Materials

The following supporting information can be downloaded at https://www.mdpi.com/article/10.3390/plants14020165/s1: S1: MPM-Pb 16084—Tridimensional model of fragment of chert block with fern rhizomatous stem in life position and associated conifer remains (e.g., roots, wood), S2: MPM-Pb 16085—Tridimensional model of fragment of chert block (counterpart of MPM-Pb 16084) with fern rhizomatous stem in life position and associated conifer remains (e.g., roots, wood). S3: MPM-Pb 16086—Tridimensional model of fragment of chert block with three fern rhizomatous stems in life position. S4: MPM-Pb 16087—Tridimensional model of fragment of chert block with a fern rhizomatous stem in life position. S5: MPM—Pb 16090—Tridimensional model of fragment of chert block with fern rhizomatous stems in life position. S6: MPM-Pb 16093—Tridimensional model of fragment of chert block with a fern rhizomatous stem in life position.
