# Supplementary material for: Jurassic Osmundaceous Landscapes in Patagonia: Exploring the Concept of Ecological Stasis in the Deseado Massif, Argentina
Source: Plants (Basel). 2025 Jan 8;14(2):165. doi: 10.3390/plants14020165 (PMC11768899; doi:10.3390/plants14020165)
Supplement: Supplementary file 1 [file plants-14-00165-s001.zip › plants-3357530-Supplementary material.pdf]

Supplementary material for

In-situ Osmundaceous ferns-dominated landscapes in the Jurassic of Patagonia: a case of ecological stasis from the Deseado Massif, Santa Cruz, Province, Argentina

Juan L. García Massini[1,5], Giovanni C. Nunes[2, 5], Agustina Yañez[3,5]*, Ignacio H. Escapa[2,5], Diego Guido[4,5]

[1] Centro Regional de Investigaciones Científicas y Transferencia Tecnológica (CRILAR), Provincia de La Rioja, UNLaR, SEGEMAR, UNCa, CONICET, Anillaco, Provincia de La Rioja, 5301, Argentina.
[2] Museo Paleontológico Egidio Feruglio, Avenida Fontana 140, Trelew, Provincia del Chubut, 9100, Argentina.
[3] Museo Argentino de Ciencias Naturales "Bernardino Rivadavia", Av. Ángel Gallardo 470, Ciudad de Autónoma de Buenos Aires, C1405DJR, Argentina.
[4] Facultad de Ciencias Naturales y Museo, Universidad Nacional de La Plata, Instituto de Recursos Minerales (UNLP-CICBA), La Plata, Provincia de Buenos Aires, 1900, Argentina.
[5] Consejo Nacional de Investigaciones Científicas y Técnicas, Godoy Cruz 2290, Ciudad Autónoma de Buenos Aires, C1425FQB, Argentina.
*Correspondence: gugu@macn.gov.ar

Tridimensional models of selected chert samples preserving one to several Osmundaceae fern individuals in life position.
Models were obtained following the methodology described in the Materials and Methods section.

3D-scanned materials:
S1. MPM-Pb 16084 — Fragment of chert block with fern rhizomatous stem in life position and associated conifer remains (e.g., roots, wood).
S2. MPM-Pb 16085 — Fragment of chert block (counterpart of MPM-Pb 16084) with fern rhizomatous stem iN life position and associated conifer remains (e.g., roots, wood).
S3. MPM-Pb 16086 — Fragment of chert block with three fern rhizomatous stems in life position.
S4. MPM-Pb 16087 — Fragment of chert block with a fern rhizomatous stem in life position.
S5: MPM-Pb 16090 — Fragment of chert block with fern rhizomatous stems in life position.
S6: MPM-Pb 16093 — Fragment of chert block with a fern rhizomatous stem in life position.

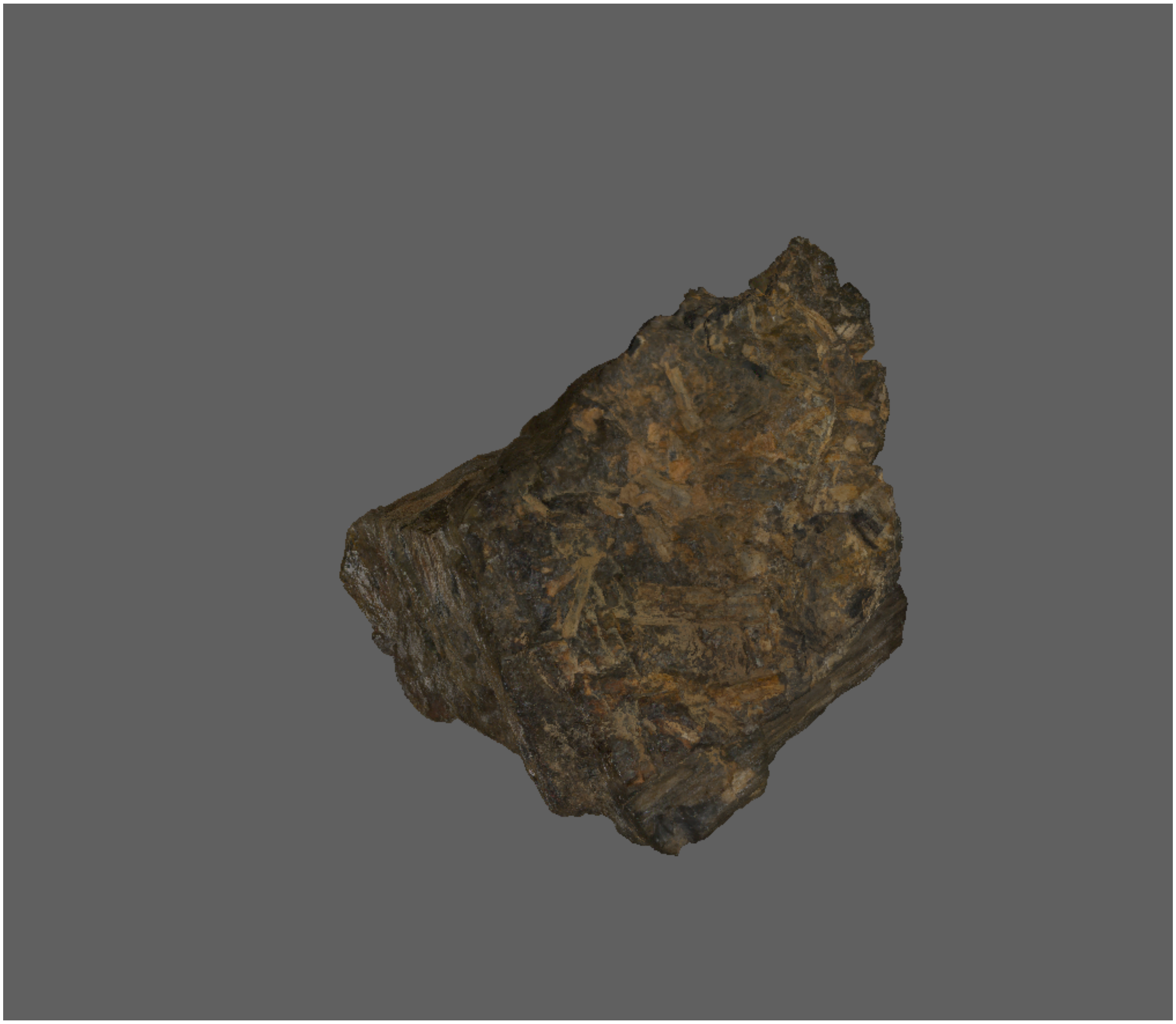

S1. MPM-Pb 16084

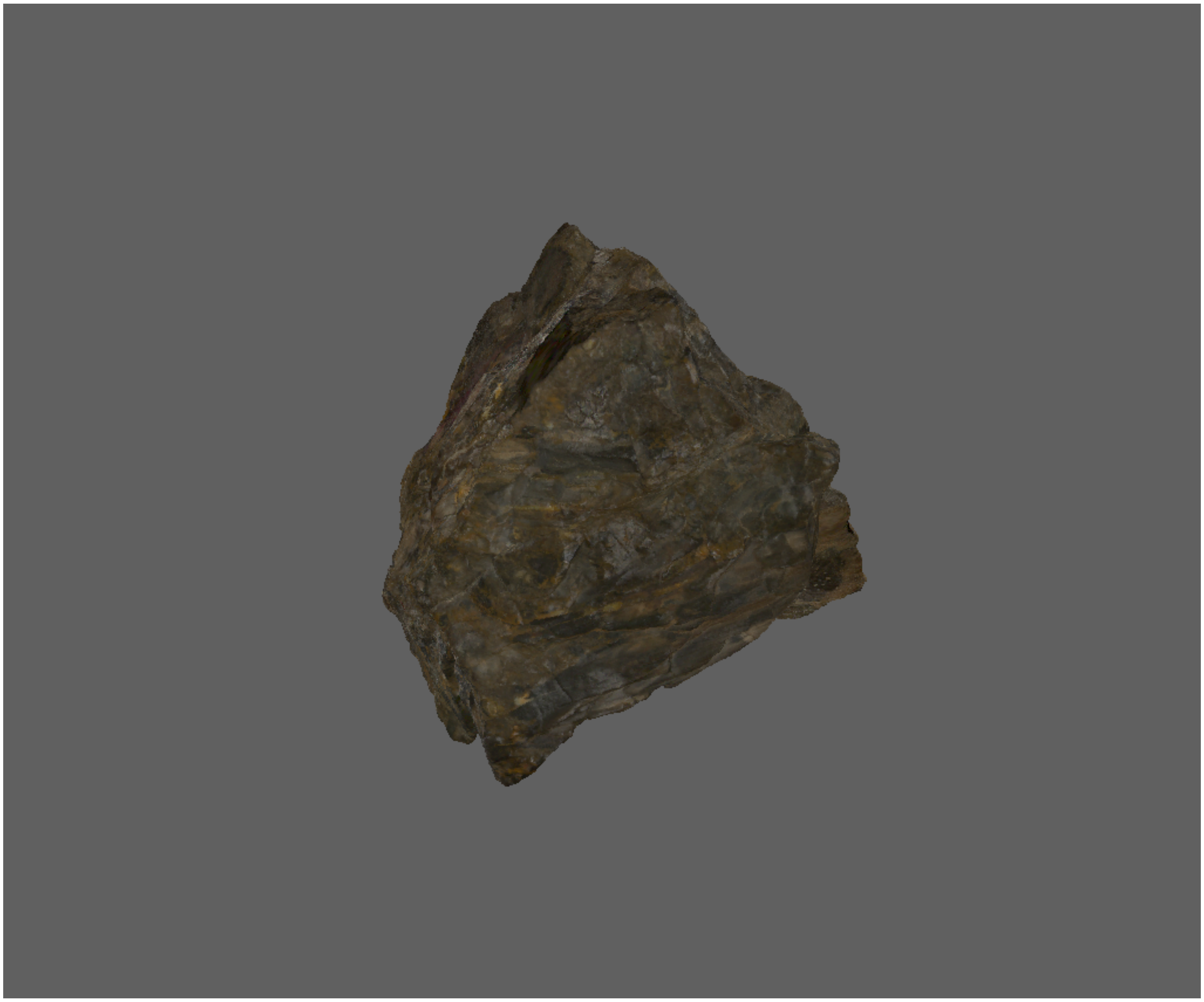

S2. MPM-Pb 16085

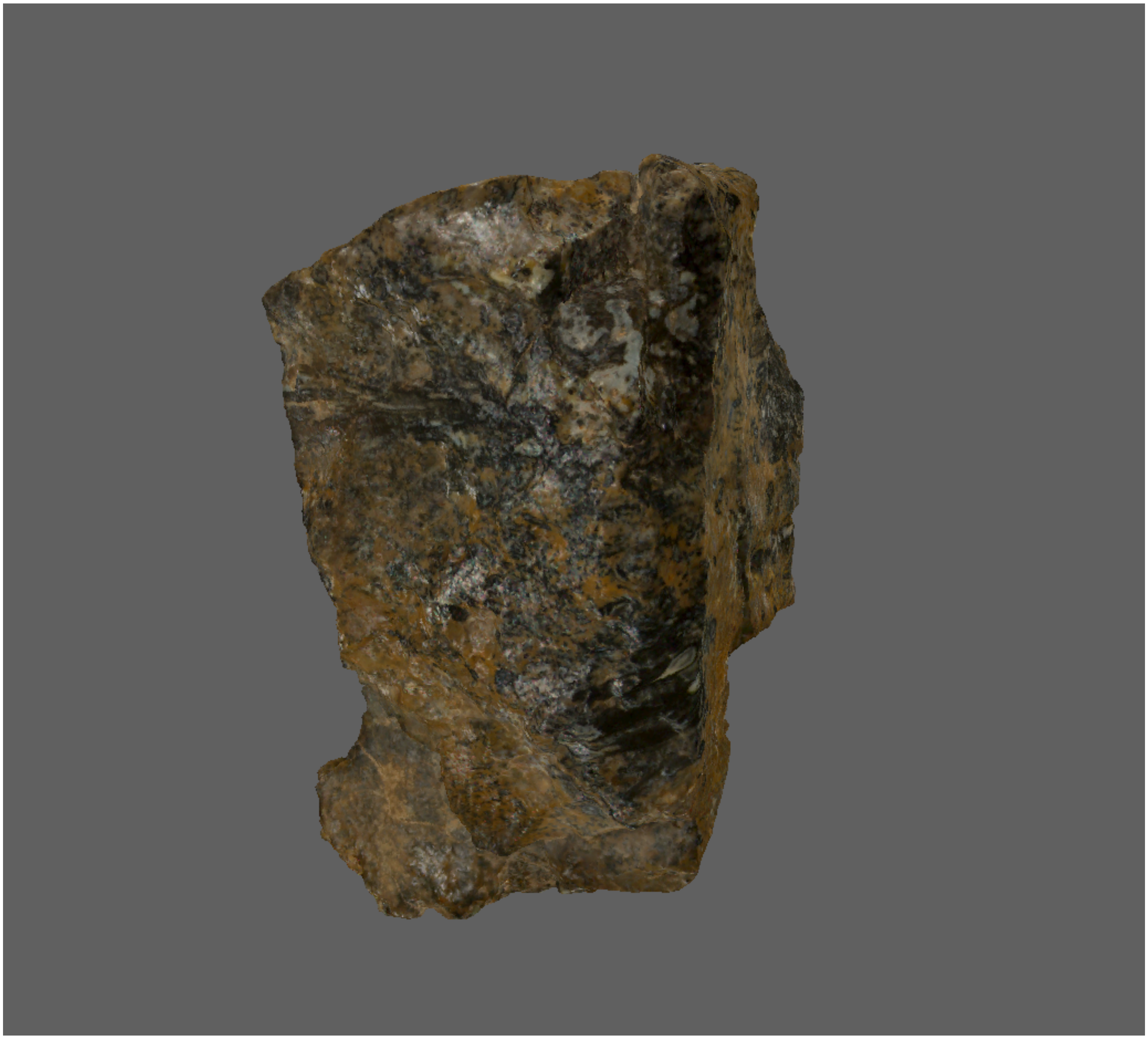

S3. MPM-Pb 16086

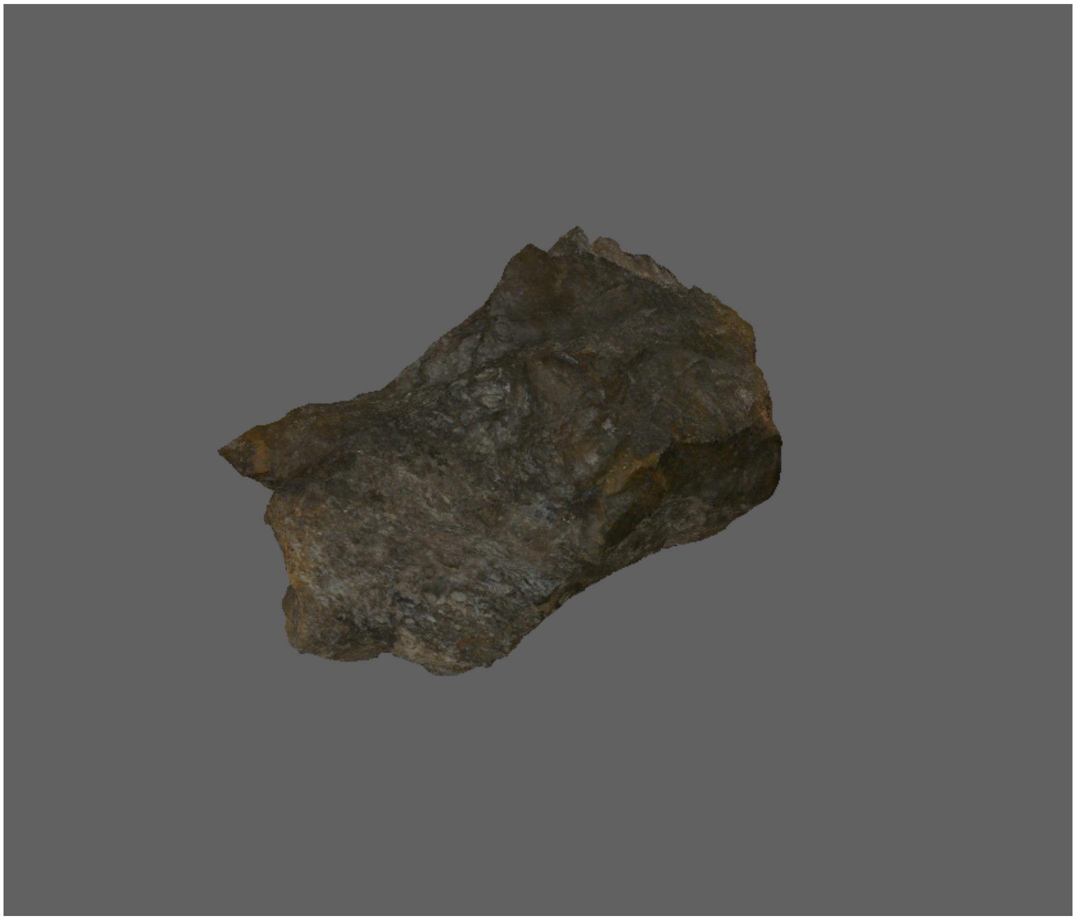

S4. MPM-Pb 16087

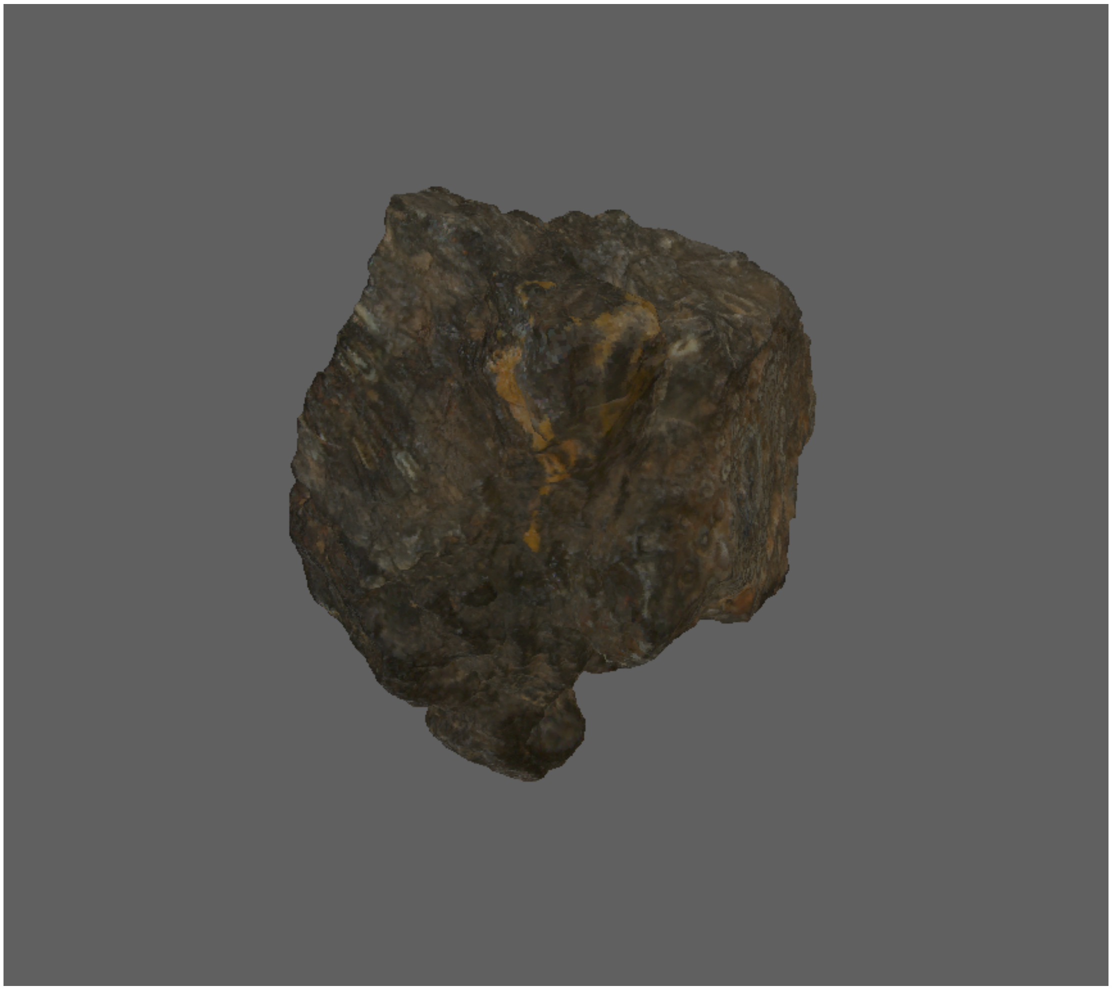

S5. MPM-Pb 16090

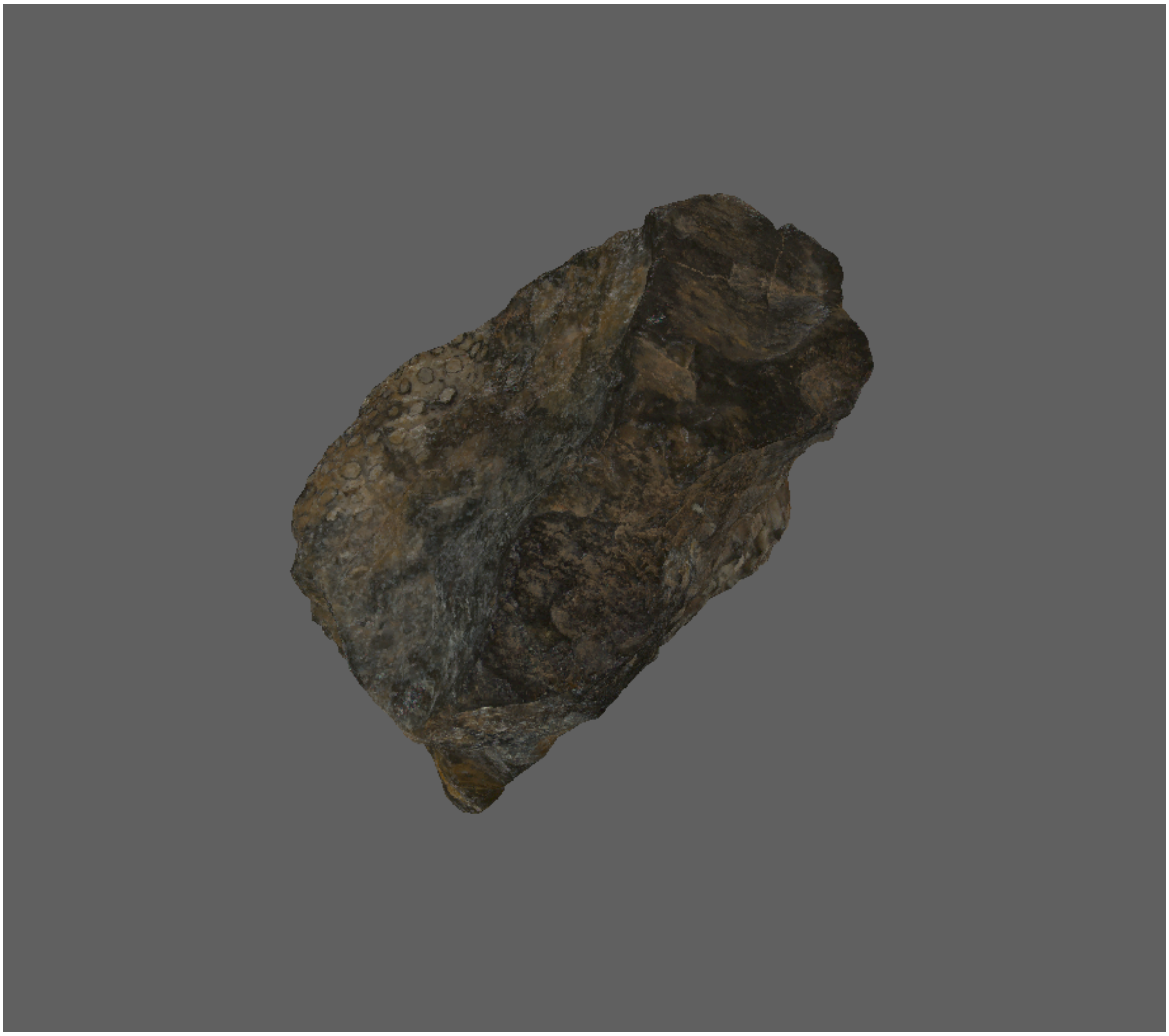

S6. MPM-Pb 16093
